# Designing a synthetic microbial community devoted to biological control: The case study of Fusarium wilt of banana

**DOI:** 10.3389/fmicb.2022.967885

**Published:** 2022-08-05

**Authors:** Maria Isabella Prigigallo, Carmen Gómez-Lama Cabanás, Jesús Mercado-Blanco, Giovanni Bubici

**Affiliations:** ^1^Istituto per la Protezione Sostenibile delle Piante, Consiglio Nazionale delle Ricerche, Bari, Italy; ^2^Departamento de Protección de Cultivos, Instituto de Agricultura Sostenible, Agencia Estatal Consejo Superior de Investigaciones Científicas, Córdoba, Spain

**Keywords:** *Fusarium oxysporum* f. sp. *cubense*, biological control agents, microbial consortia, beneficial microbes, *Pseudomonas chlororaphis* subsp. *piscium*, *Bacillus velezensis*, *Trichoderma virens*

## Abstract

*Fusarium oxysporum* f. sp. *cubense* (*Foc*) tropical race 4 (TR4) is threatening banana production because of its increasing spread. Biological control approaches have been widely studied and constitute interesting complementary measures to integrated disease management strategies. They have been based mainly on the use of single biological control agents (BCAs). In this study, we moved a step forward by designing a synthetic microbial community (SynCom) for the control of Fusarium wilt of banana (FWB). Ninety-six isolates of *Pseudomonas* spp., *Bacillus* spp., *Streptomyces* spp., and *Trichoderma* spp. were obtained from the banana rhizosphere and selected *in vitro* for the antagonism against *Foc* TR4. In pot experiments, a large community such as SynCom 1.0 (44 isolates with moderate to high antagonistic activity) or a small one such as SynCom 1.1 (seven highly effective isolates) provided similar disease control (35% symptom severity reduction). An *in vitro* study of the interactions among SynCom 1.1 isolates and between them and *Foc* revealed that beneficial microorganisms not only antagonized the pathogen but also some of the SynCom constituents. Furthermore, *Foc* defended itself by antagonizing the beneficial microbes. We also demonstrated that fusaric acid, known as one of the secondary metabolites of *Fusarium* species, might be involved in such an interaction. With this knowledge, SynCom 1.2 was then designed with three isolates: *Pseudomonas chlororaphis* subsp. *piscium* PS5, *Bacillus velezensis* BN8.2, and *Trichoderma virens* T2C1.4. A non-simultaneous soil application of these isolates (to diminish cross-inhibition) delayed FWB progress over time, with significant reductions in incidence and severity. SynCom 1.2 also performed better than two commercial BCAs, BioPak^®^ and T-Gro. Eventually, SynCom 1.2 isolates were characterized for several biocontrol traits and their genome was sequenced. Our data showed that assembling a SynCom for biocontrol is not an easy task. The mere mixtures of antagonists (e.g., SynCom 1.0 and 1.1) might provide effective biocontrol, but an accurate investigation of the interactions among beneficial microorganisms is needed to improve the results (e.g., SynCom 1.2). SynCom 1.2 is a valuable tool to be further developed for the biological control of FWB.

## Introduction

Fusarium wilt of banana (FWB), caused by the soil-borne fungus *Fusarium oxysporum* f. sp. *cubense* (*Foc*), is one of the most devastating diseases of the banana crop and occurs in all banana-producing countries (Dita et al., 2018). In the 1950s’, an epidemic caused by *Foc* race 1 (R1) was tackled by the use of the resistant variety Cavendish, which replaced the susceptible ‘Gros Michel’ (Ploetz, 2006). In the 1990s’, *Foc* tropical race 4 (TR4) emerged as a new menace against the banana crops (Ploetz, 2006). Due to its increasing spread (Dita et al., 2018; Pegg et al., 2019), it still deserves the attention of scientists. Nowadays, although promising germplasm is available (Li et al., 2015; Bubici et al., 2019, and references within; Zorrilla-Fontanesi et al., 2020), no *Foc* TR4-resistant varieties have been implemented in the banana production system.

Historically, biological control has been widely studied and is nowadays considered a sustainable and complementary measure within integrated disease management frameworks (O’Brien, 2017; He et al., 2021). A comprehensive literature review has shown that half of the studies on FWB have dealt with disease management, with papers mainly focused on biological control and breeding (Bubici et al., 2019). In that review article, studies involving biological control agents (BCAs) against *Foc* in *in vitro* assays, pot experiments, and in the field have been compiled. They showed that good biocontrol effectiveness and yield increases have been achieved so far. For example, the combination of an organic fertilizer with *Bacillus amyloliquefaciens* NJN-6 significantly decreased FWB incidence by 68.5% and doubled the yield in the field (Xue et al., 2015). Another *B. amyloliquefaciens* strain, W19, also provided effective biocontrol when combined with an organic fertilizer (Wang et al., 2016). Applications of a *Pseudomonas fluorescens* Pf-1 formulation reduced wilt incidence by 80.6% in the field (Raguchander et al., 2000). Sometimes, the origin of those BCAs has not been specified. In the search for BCAs, the selection of microorganisms from the environment where they are intended to be applied implies adaptive advantages for these microorganisms. Moreover, it is broadly accepted that disease-suppressive soils enhance the chances of identifying effective BCAs (Xue et al., 2015).

Often based on the application of single strains, biological control of plant diseases has been limited by inconsistent results over years and environments (Mitter et al., 2019). Recently, a trend toward the use of microbial consortia, or synthetic microbial communities (SynComs), has surfaced with the ambition of higher effectiveness, versatility, multifunctionality, and stability in the environment (Czajkowski et al., 2020). Nevertheless, if the success of a single strain is difficult to predict in diverse agricultural systems, this is even more complicated for microbial consortia because of complex interactions among their constituents (Barea et al., 2005; Mitter et al., 2019; Trivedi et al., 2020). A SynCom is an artificial assembly of microorganisms that mimics a natural community though with reduced complexity (Grosskopf and Soyer, 2014). Much research on SynCom has been made for biotechnological purposes, and special attention has been paid to several aspects, including the principles for designing SynCom, optimization of the composition for a more efficient functionality (e.g., production of certain molecules), compatibility among strains, etc. (Smith, 2011; Johns et al., 2016; de Souza et al., 2020). The literature agrees that microbial consortia outperform formulations based on single strains, both for biotechnological tasks (Faust, 2019; Mauri et al., 2020; Sgobba and Wendisch, 2020) and biocontrol (Whipps, 2001; Thakkar and Saraf, 2015; Bradáčová et al., 2019; Minchev et al., 2021). One of the few examples of microbial consortia used against FWB was the combination of two endophytes, *B. subtilis* EPB56 and EPB10, and the rhizobacterium *P. fluorescens* Pf1, which gave 78% disease reduction (Kavino and Manoranjitham, 2018). Another small consortium composed of *P. fluorescens* Pf1 and *B. subtilis* TRC 54 was delivered to banana plants together with a botanical formulation and reduced FWB incidence significantly under greenhouse (64%) and field conditions (75%) (Akila et al., 2011).

Designing microbial consortia is challenging, and successful examples are rare (Faust, 2019). This is particularly true in agriculture, where the research is at an earlier stage. The strains to be included in a given SynCom need in-depth studies aiming to obtain additive or synergistic positive effects and to avoid cross-inhibition among the strains or negative effects on the plants. Consequently, the overall biocontrol is the result of the combination of tasks performed by each of the constituents. With a poor knowledge of the interaction taking place among the components of a consortium, the simple mixture of effective BCAs might result in a scarce biocontrol.

The main objectives of this work were: (a) to establish a collection of potential BCAs (*Pseudomonas* spp., *Bacillus* spp., *Streptomyces* spp., and *Trichoderma* spp.) against FWB obtained from the banana rhizosphere; (b) to identify possible difficulties in designing SynComs to be used for biological control; and (c) to develop a SynCom effective against FWB under controlled conditions and worthy of future field testing (viz., effective under controlled conditions and/or outperforming commercial BCAs). We hypothesized that a large community of beneficial microorganisms can cope with a plant pathogen like *Foc* better than small consortia (e.g., two strains) or single BCAs. This theory comes from the evidence that the manipulation of soil microbiota in favor of the plant-beneficial portion can provide advantages to the crops (Bender et al., 2016; Banerjee et al., 2019). Also, we hypothesized that precise knowledge of microbe-microbe interactions among strains is crucial for designing a SynCom (Smith, 2011; de Souza et al., 2020) because they are not always unidirectional (e.g., BCAs antagonize *Foc*). We show evidence that our SynCom isolates not only antagonized the pathogen but also some of the SynCom constituents, while the pathogen actively defended itself by hindering the growth of the SynCom isolates. We encourage this type of study, neglected in the literature so far, for future research aimed to develop microbial consortia for biocontrol.

## Materials and methods

**FIGURE 1:**
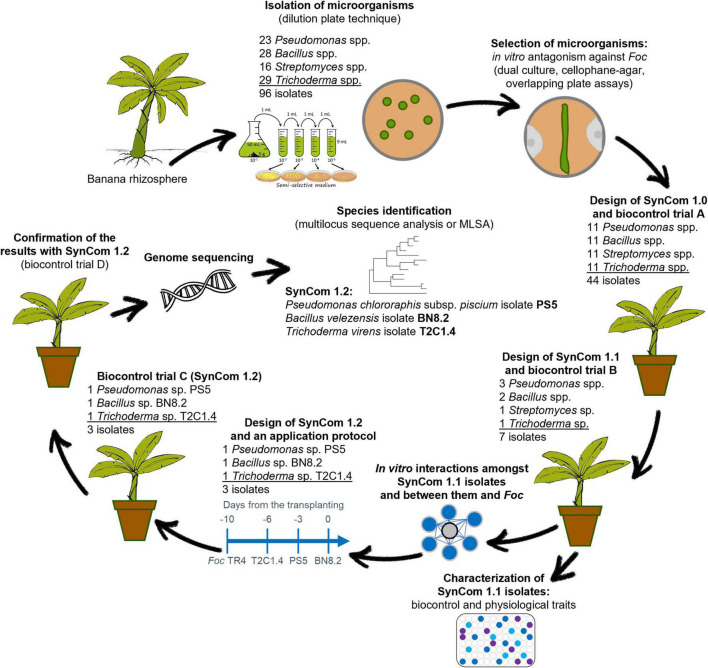
Schematic representation of the steps of our research which led to the development of a synthetic microbial community namely SynCom 1.2 effective against *Fusarium oxysporum* f. sp. *cubense* (*Foc*) tropical race 4 (TR4) starting from the isolation of microorganisms from the banana rhizosphere.

The steps of the present research are schematized in Figure 1. A series of *in vitro* and *in planta* experiments were performed to develop a SynCom effective against *Foc* TR4 starting from the isolation of microorganisms from the banana rhizosphere.

### Isolation of microorganisms

Forty-three rhizospheric soil samples taken from several banana farms on Tenerife Island (see Gómez-Lama Cabanás et al., 2021, for details) were used for the isolation of beneficial microorganisms. Potential antagonists were successfully isolated from 35 (reported in Supplementary Table S1) out of the 43 samples.

Using the dilution plate technique, microorganisms were isolated on agar media semi-selective for four taxa notoriously harboring BCAs: *Pseudomonas* spp., *Bacillus* spp., *Streptomyces* spp., and *Trichoderma* spp. Briefly, 5 g of soil were suspended in 50 mL physiological solution (8.5 g NaCl, 2 drops Tween 20, 1 g agar, 1 L H_2_O) and stirred for 30 min. This suspension was used for serial ten-fold dilutions and then plated in triplicate on the following semi-selective agar media. King’s B (KB) (King et al., 1954) amended with 100 mg L^–1^ cycloheximide, 50 mg L^–1^ ampicillin, 12.5 mg L^–1^ chloramphenicol (Simon and Ridge, 1974), and 5 mg L^–1^ pentachloronitrobenzene (PCNB) (Gamliel and Katan, 1991) was used for *Pseudomonas* spp. Nutrient agar (NA; 8 g L^–1^ nutrient broth, 16 g L^–1^ agar) was used for *Bacillus* spp. The selectivity for this genus was ensured by the incubation of soil suspension at 80°C for 10 min immediately before the plating (Klement et al., 1990). Starch-casein-KNO_3_ (SCPN) agar was used for *Streptomyces* spp. (Küster and Williams, 1964) and *Trichoderma harzianum*-selective medium (THSM) for the isolation of *Trichoderma* spp. (Williams et al., 2003). After 7 days of incubation at 27°C in the dark, 5–10 non-coalescing colonies (preferably with diverse morphotypes) per soil sample were randomly selected and transferred to Luria-Bertani agar (LBA; for *Pseudomonas* spp. and *Bacillus* spp.) or potato dextrose agar (PDA; for *Streptomyces* spp. and *Trichoderma* spp.) plates for checking the purity. At this stage, isolates were identified at the genus level only upon colony morphology and microscopic characteristics. Eventually, 96 isolates (23 *Pseudomonas* spp., 28 *Bacillus* spp., 16 *Streptomyces* spp., and 29 *Trichoderma* spp.) were obtained and their cell/spore/conidial suspensions were stored at –80°C in 1 mL-cryovials containing 25, 15, or 10% glycerol (in water) for *Streptomyces* spp., other bacteria, or fungi, respectively.

### *In vitro* antagonistic activity of microorganisms against *Fusarium oxysporum* f. sp. *cubense*

The antagonistic activity of the 96 isolates was evaluated against *Foc* TR4 strain NRRL36114 (= CBS 102025) (Dita et al., 2010), which was obtained from the Westerdijk Fungal Biodiversity Institute (Utrecht, Netherlands). The dual culture method was used to evaluate the effect of diffusible metabolites and volatile compounds of *Pseudomonas* spp., *Bacillus* spp., and *Streptomyces* spp. The dual culture assay was not used for *Foc* and *Trichoderma* spp. because of their rapid growth. Hence, for these fungi, the cellophane-agar technique and the overlapping plate method were implemented to evaluate the potential antagonistic effects of diffusible metabolites and volatile compounds, respectively.

The dual culture method was carried out according to Bubici et al. (2013). Briefly, a 10 μL-drop of cell/spore suspensions (10^7^ cells or spores mL^–1^) of bacteria was streaked along a diameter of a 90 mm Petri plate containing PDA (Condalab, Madrid, Spain). After 3 days of incubation at 27°C in the dark, two 6 mm-agar plugs of *Foc* TR4 (taken from the edge of a 7-day-old colony) were placed at the edges of the orthogonal diameter of the plate. Plates inoculated with *Foc* but not with bacteria served as control. Four-replicated plates were incubated for 7 days at 27°C in the dark.

In the cellophane-agar method, an autoclaved 100 mm-cellophane disk was placed on the PDA surface in a 90 mm Petri plate. A 6 mm-agar plug of the *Trichoderma* spp. isolates (taken from the edge of 7 days old colonies) was inoculated on the cellophane disk in the center of the plate. After 72 h at 27°C in the dark, the cellophane disk containing the tested strain was removed and five 6-mm agar plugs colonized by *Foc* TR4 were placed in the center of plates and at the edges of two orthogonal diameters. By using this setup, *Trichoderma* spp. isolates grown over the cellophane disks (72 h at 27°C in the dark) released metabolites into the agar without physical contact of the hyphae with the culturing medium. Then, a 6-mm agar plug of *Foc* TR4 (taken from the edge of a 7-day-old colony) was placed in the center of the plates (four replicates) and incubated for 7 days at 27°C in the dark.

The overlapping plate assay was performed according to Prigigallo et al. (2021). A 6 mm-agar plug of *Trichoderma* spp. isolates (taken from the edge of 7 days old colonies) was inoculated in the center of a 9 mm PDA plate (bottom plate), which was then incubated at 27°C in the dark for 2 days. Then, another plate (upper plate) was inoculated with a 6 mm-agar plug of *Foc* TR4 (taken from the edge of a 7-day-old colony) and overlapped upside down on the bottom plate (both plates without the lid). The two plates were sealed with Parafilm M^®^ to avoid the escape of volatile compounds and incubated at 27°C in the dark for 7 days. During the incubation, volatile compounds emitted by *Trichoderma* spp. saturated their headspace and the potential impact on the growth of *Foc* was evaluated.

At the end of each assay, photos were taken under standardized conditions of light and distance of the plate from the camera objective. Image analysis was conducted using ImageJ version 1.53m (Schneider et al., 2012) to calculate the area of *Foc* colonies and its reduction percentage in bacteria- or *Trichoderma*-inoculated plates compared to control. Heatmaps were also generated to represent the *Foc* inhibition level caused by the potential antagonists.

### Design of synthetic microbial communities (SynComs) and their evaluation for the biocontrol

Three SynComs namely SynCom 1.0, 1.1, and 1.2 were designed, each with a different number of isolates. Four consecutive pot experiments were carried out to identify the most effective SynCom. *Foc* TR4 pressure was progressively decreased from the first (A) to the last (D) biocontrol trial to increase the biocontrol efficiency and recall more natural (field) conditions. The initial *Foc* TR4 inoculum was 10^7^ colony-forming units or CFU g^–1^ of soil in biocontrol trial A, 5.10^5^ in biocontrol trials B and C, and 10^4^ CFU g^–1^ in biocontrol trial D. This approach was used to sequentially evaluate the SynComs, and only the most effective SynCom (1.2) was evaluated in two independent experiments (C and D). Steam-sterilized soil and tissue-culture micropropagated banana plantlets were used in all pot experiments to avoid possible and unpredictable interference of natural microbiota with SynComs.

### Biocontrol trial A: SynCom 1.0

**FIGURE 2:**
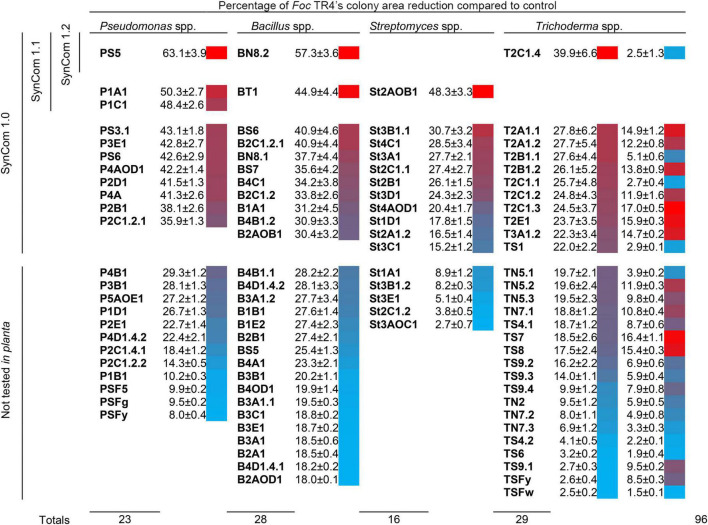
Antagonistic activity of 96 microbial isolates obtained from the banana rhizosphere against *Fusarium oxysporum* f. sp. *cubense* (*Foc*) tropical race 4 (TR4). Means of four replicates, standard error, and heatmap are shown. Data for *Pseudomonas* spp., *Bacillus* spp., and *Streptomyces* spp. were obtained from dual culture assays (which allow evaluating the effect of both agar-diffusible metabolites and volatile compounds), and those for *Trichoderma* spp. from cellophane-agar (first column; only agar-diffusible metabolites) and overlapping plate methods (second column; only volatile compounds). Isolates included in the synthetic microbial communities SynCom 1.0, 1.1, and 1.2 are also indicated.

SynCom 1.0 was designed with 44 isolates, viz. the best 11 isolates of the four genera based on the *in vitro* antagonism assays (Figure 2). We preferred to have an equal number of isolates per genus for equilibrium among the genera. The selected isolates yielded more than 30% (for *Pseudomonas* spp. and *Bacillus* spp.), 15% (for *Streptomyces* spp.), and 20% (for *Trichoderma* spp.) *in vitro* inhibition of *Foc* TR4. The rationale of this choice was to include isolates with some (though not very high) detectable antagonistic activity against *Foc*.

The trial was conducted in 1.4 L plastic pots containing steam-sterilized soil inoculated with *Foc* TR4, 7 days before transplanting (dbt), and SynCom 1.0, three dbt. The *Foc* inoculum was prepared in a mixture of soil:peat:sand:cornmeal 10:3:1:1. This substrate was autoclaved (121°C, 1 atm, 1 h) twice at a 24 h interval in 1 L aluminum trays with a lid. Then, the substrate was inoculated with 10 mL conidial suspension (10^4^ conidia mL^–1^) of *Foc* TR4 and incubated at 27°C for 21 days. At the end of the incubation period, the inoculum concentration was determined by the dilution plate technique on PDA starting from 5 g inoculum. The inoculum was mixed with soil to reach 10^7^ CFU g^–1^ of soil. The inocula of SynCom isolates were prepared individually. *Pseudomonas* spp. and *Bacillus* spp. isolates were grown in 50 mL tubes containing 20 mL LB broth and incubated at 27°C at 250 rpm for 3 days. Then, the cultures were centrifuged at 8,000 × *g* for 10 min, washed with sterile distilled water (i.e., resuspension and centrifugation), and resuspended in sterile distilled water. *Streptomyces* spp. and *Trichoderma* spp. inocula were prepared by washing (sterile distilled water) PDA plates with the agar surface completely colonized, and previously incubated at 27°C in the dark for 21 or 7 days, respectively. The cell, spore, or conidial concentration was estimated using a Thoma cell counting chamber. Finally, the inocula were mixed with the soil to reach ca. 10^6^ cells g^–1^ of soil per isolate, viz. ca. 10^7^ cells g^–1^ of soil per genus. Sterile distilled water was used to inoculate the control treatment.

Tissue-culture micropropagated banana plantlets variety ‘Gran Enana’ (obtained from Vitropic, Saint-Mathieu-de-Tréviers, France) were acclimated for 4 weeks in 0.2 L pots containing commercial potting soil, placed in a glasshouse at 27°C and 80% relative humidity (100% during the first week). Plants were irrigated as needed and fertilized once a week with Hoagland’s solution (Hoagland and Arnon, 1950). After this hardening period, the plantlets were used for the biocontrol trial.

The treatments were the following: untreated control, *Foc* TR4, SynCom 1.0, and *Foc* TR4 + SynCom 1.0. They were arranged in a randomized complete block design with three replicates, each with five plants. Plants were grown in a conditioned glasshouse at 27°C, with 80% relative humidity, and natural lighting, under strict containment conditions to avoid the accidental escape of the pathogen. Plants were irrigated as needed and fertilized once a week with Hoagland’s solution, but no phytosanitary treatments were made.

Fusarium wilt of banana symptoms were evaluated weekly until 28 days post-transplanting (dpt) when FWB incidence was near 100% in the control and/or the first senescence symptoms occurred. Symptom severity was assessed according to the following 0–4 scale: 0 = no symptoms; 1 = yellowing of one leaf; 2 = yellowing of 2–3 leaves; 3 = yellowing and wilting of several leaves; 4 = yellowing, wilting, and necrosis of several leaves. Disease incidence was expressed as the percentage of diseased plants. At the end of the experiment, the vascular browning was evaluated on the cross-section of the corm according to the following 0–4 scale: 0 = no discoloration; 1 = 1–10%; 2 = 11–25%; 3 = 26–50%; 4 = 51–100% of the section affected by vascular browning.

The kinetics of the *Foc* TR4 population in the soil was determined using the aforementioned dilution plate technique. At the transplanting time, and weekly afterward, 5 g of soil were taken from each pot at 5 cm-depth and used to plate their 10-fold dilutions on Komada’s semi-selective medium (Komada, 1975). Four-replicated plates were incubated at 27°C in the dark for 3 days when *F. oxysporum* colonies were distinctly visible. Data were expressed as CFU per gram of dried soil, as determined every time after drying a soil aliquot at 80°C until constant weight. Since the agar medium was semi-selective for *F. oxysporum*, all the colonies growing on this medium were presumably *Foc* TR4, though they were expressed as *Fusarium* spp. in the graphs. The same approach, but with the semi-selective media used for the isolation, was adopted to determine the population kinetics of the beneficial microorganisms in the soil. In the same way, the abundance of *Foc* TR4 and beneficial microbes was evaluated in the corm at 28 dpt. For this purpose, the corm was deprived of leaves, surface-sterilized (0.5% NaClO for 2 min), rinsed in sterile distilled water, and a 5 g portion was macerated with pestle a mortar to be used in the dilution plate procedure. Data were expressed as CFU per gram of fresh plant tissue.

Leaf chlorophyll content of all plants was measured over time on the last fully expanded leaf using atLEAF CHL PLUS (FT green LLC, Wilmington, DE, United States).

### Biocontrol trial B: SynCom 1.1

SynCom 1.1 was conceived as a community exclusively composed of highly antagonistic isolates (at least 75% of the maximum value of *in vitro Foc* inhibition per taxon). This consortium included three *Pseudomonas* spp. isolates (P1A1, P1C1, and PS5), two *Bacillus* spp. isolates (BN8.2 and BT1), one *Streptomyces* sp. isolate (St2AOB1), and one *Trichoderma* sp. isolate (T2C1.4) (Figure 2). This experiment was conducted as the previous one but with a lower initial *Foc* TR4 inoculum (5.10^5^ CFU g^–1^ soil) in an attempt to increase the biocontrol efficiency. This experiment ended 35 dpt when FWB incidence was near 100% in the control and/or the first senescence symptoms occurred.

To further ascertain whether the effect of SynCom 1.1 against *Foc* TR4 in the soil depended on the pathogen inoculum density, another experiment was carried out with pots containing soil but without plants. *Foc* TR4 was inoculated into steam-sterilized soil (1.4 L-plastic pots) as previously described but using several inoculum densities namely 10^3^, 10^5^, and 10^7^ CFU g^–1^. SynCom 1.1 was also inoculated as described above. Pots were maintained in the greenhouse as in the biocontrol trials. Over 32 days, *Foc* TR4 populations in treated and untreated soils were monitored using the dilution plate technique and the semi-selective agar medium.

### Characterization of selected microorganisms and optimization of the SynCom

#### Biocontrol traits and physiological characterization of SynCom 1.1 isolates

Several traits associated with antagonism and/or plant growth promotion were evaluated to obtain an overview of the attitude to biocontrol of our isolates. Only seven isolates, which then composed SynCom 1.1 (see below), were included in these assays as highly effective antagonists of *Foc* TR4. Several other bacteria routinely used in our laboratory were included in these assays as biocontrol reference strains (Montes-Osuna et al., 2021a): *Pseudomonas* sp. PICF6 (Montes-Osuna et al., 2021b), *P. simiae* PICF7 (Montes-Osuna et al., 2021a), *Pseudomonas* sp. PIC25 (Gómez-Lama Cabanás et al., 2018a), which were isolated from olive roots, and *Pantoea agglomerans* IAS-B-1036, *Chryseobacterium* sp. IAS-B-370, *P. chlororaphis* IAS-B-1013, and isolate IAS-B-717, originating from the banana root endosphere (Gómez-Lama Cabanás et al., 2021). Protease, catalase, phosphatase, phytase, β-glucosidase, 1-aminocyclopropane-1-carboxylate (ACC) deaminase, and amylase activities, as well as the production of 2,3-butanediol, hydrogen cyanide (HCN), and siderophores, were assessed as previously described (Gómez-Lama Cabanás et al., 2018a and references therein). Ammonia (NH_3_) production was determined using Nessler’s reagent as described by Prigigallo et al. (2021). Moreover, the capability of three *Pseudomonas* spp. isolates (P1A1, P1C1, and PS5, later included in SynCom 1.1) to metabolize 71 carbon sources, and the sensitivity to 23 chemicals were determined, using the GEN III MicroPlate™ (Biolog, Hayward, CA, United States) system according to the manufacturer’s instructions. The phenotypic pattern was used in the Biolog’s Microbial Identification Systems software for the species identification. These assays were conducted in duplicate.

#### *In vitro* interactions among SynCom 1.1 isolates

The effects of agar-diffusible metabolites and volatile compounds produced by the beneficial microorganisms against each other were investigated by the cellophane-agar method and overlapping plate assay, respectively. Only seven isolates, which then composed SynCom 1.1 (see below), were included in these experiments because of their high effectiveness against *Foc* TR4.

The assays were conducted as described above but with some modifications. On the cellophane disk, bacterial isolates were inoculated by a 100 μL cell/spore suspension (10^7^ cells or spores mL^–1^) placed in the center of the plate. This implied the release of the metabolites in the inoculation point and a decreasing concentration gradient toward the plate edge. Such a gradient was similarly created in the *Trichoderma* plates because the fungus was inoculated by a 6 mm-agar plug. After the cellophane removal, a 20 μL-drop (10^7^ cells or spores mL^–1^) was streaked along two orthogonal diameters of the plate.

In the overlapping plate assay, bacterial isolates were streaked all over the agar surface in the bottom plate (100 μL of 10^7^ cells or spores mL^–1^) and streaked along two orthogonal diameters in the upper plate (20 μL-drop of 10^7^ cells or spores mL^–1^). Microorganisms in the bottom plate were inoculated 2 days (seven for *Streptomyces* sp. St2AOB1) before the inoculation of the upper plate. *Trichoderma* sp. T2C1.4 was always inoculated by a 6 mm-agar plug. Bacteria were grown either on PDA or LBA, and the fungus only on PDA.

Four-replicated plates were prepared, and plates without the microbial donor of metabolites or volatiles served as controls. The plates were incubated for 7 days at 27°C in the dark, and then inspected visually and photographed.

#### Effects of *Fusarium oxysporum* f. sp. *cubense* and fusaric acid on SynCom 1.1 isolates

The cellophane agar method and the overlapping plate assay described above were also used to evaluate the effect of metabolites produced by *Foc* against the seven isolates constituting SynCom 1.1. Besides *Foc* TR4, *Foc* R1 (NRRL36110 = CBS102021; obtained from the Westerdijk Fungal Biodiversity Institute) (Dita et al., 2010) was also included in these experiments.

Fusaric acid (FA) is a well-known pathogenicity factor of *Fusarium* species (Liu et al., 2020). To evaluate its role in the activity of *Foc* against the beneficial microorganisms, the effective concentrations able to reduce the microbial growth by 50% (EC_50_) were determined. FA (CAS 536-69-6, 99% pure powder, Acros Organics, Morris Plains, NJ, United States) was dissolved in sterile potato dextrose broth (PDB; Condalab, Madrid, Spain) at a concentration of 10 mM and twofold serial dilutions up to 0.078125 mM. PDB-FA solutions were distributed in sterile Primo 96 round bottom-wells microplates (ET3196, EuroClone, Pero, Milan, Italy) with 150 μL per well. PDB without FA served as control. The wells were inoculated with 30 μL microbe inoculum (10^4^ cell/spore/conidia per mL), which brought 300 CFU into the wells. Bacterial inocula were prepared from overnight LB broth cultures incubated at 27°C and 250 rpm. Then, the cultures were centrifuged at 8,000 × *g* for 10 min, washed, and resuspended in sterile distilled water. Fungal inocula were prepared by gently washing 7 days old colonies grown at 27°C in the dark. The concentration of cells, spores, or conidia was determined using the Thoma cell counting chamber under the microscope. Four replicates (wells) were used, and the microplates were incubated (27°C, 40 rpm) for 7 days. Microbial growth at 16, 24, 36, and 48 h post-inoculation was measured by the optical density at 595 nm (OD_595_) using the ELISA microplate reader Infinite^®^ F200 PRO (Tecan Trading AG, Männedorf, Switzerland).

For the *Streptomyces* sp. isolate, the assay was performed in 15 mL tubes instead of the microplates because its slow growth required an incubation time longer than that of the other tested isolates. Five mL PDB amended with FA at the abovementioned concentrations were inoculated with 100 μL of a spore suspension (10^7^ spores mL^–1^) obtained by washing a PDA plate with the whole agar surface colonized and previously incubated at 27°C in the dark for 20 days. At 7, 9, 11, and 14 days after inoculation, 150 μL cultures were transferred to the wells of a 96-well microplate for the OD_595_ measurement as described above.

### Biocontrol trials with SynCom 1.2

#### Biocontrol trial C: SynCom 1.2

SynCom 1.2 was constructed with isolates showing the highest *in vitro* antagonism against *Foc* TR4 and cross-compatibility (low or no antagonism between each dual combination of them): *Pseudomonas* sp. PS5, *Bacillus* sp. BN8.2 and *Trichoderma* sp. T2C1.4 (see the Results section for further details). Moreover, since the compatibility among these isolates was not complete, we conceived an application protocol aimed at reducing the negative effects (antagonism) of the beneficial microorganisms against each other. The three beneficial microorganisms were not inoculated simultaneously. After the inoculation of *Foc* TR4 (10 dbt), *Trichoderma* sp. T2C1.4 was firstly inoculated (6 dbt) to gain some advantage over *Pseudomonas* sp. PS5 (3 dbt), which could partially inhibit T2C1.4. Moreover, T2C1.4 was resistant to *Foc* TR4, hence it could grow without limitation in *Foc* TR4-inoculated soil. Since *Bacillus* sp. BN8.2 could potentially inhibit T2C1.4, this antagonist was inoculated by root dipping at transplanting to protect plant roots from *Foc* TR4 infections but avoiding rapid spread into to whole soil volume where the bacterium could antagonize T2C1.4 and could be inhibited by T2C1.4 and *Foc*. Inocula were prepared as described above, and the trial was conducted as the previous one but it lasted 70 days and clay pots were used. In this experiment, clay pots were used because we observed a better growth of banana plants in other our experiments.

#### Biocontrol trial D: SynCom 1.2 versus commercial biological control agents

This trial was established to confirm the results obtained with SynCom 1.2 and to compare them with those obtained with two commercial BCAs (viz., PHC BioPak^®^ and T-Gro). PHC BioPak^®^ contains several *Bacillus* spp. and one *Paenibacillus azotofixans* strain (7.5.10^9^ CFU g^–1^ each; Modern Arboriculture Institute s.r.l., Varese, Italy). T-Gro contains *Trichoderma asperellum* strain *kd* (2.10^9^ conidia g^–1^; Andermatt Biocontrol AG, Grossdietwil, Switzerland). The viability of the microbes in these products was verified before use. This experiment was carried out as the previous one with the following modifications. Five treatments were included: *Foc* TR4, SynCom 1.2_×1_, SynCom 1.2_×3_, PHC BioPak^®^, and T-Gro (all the treatments were applied in *Foc* TR4-inoculated soil). The initial *Foc* concentration was 10^4^ CFU g^–1^ soil and the trial ended at 105 dpt when FWB incidence was 100% in the control.

SynCom 1.2_×1_ treatment corresponded to the protocol used in trial C (non-simultaneous application of the isolates). In the SynCom 1.2_×3_ treatment, two additional applications of the three isolates were made at seven and 14 dpt following the SynCom 1.2_×1_ treatment. At seven and 14 dpt, the isolates were applied simultaneously by soil drenching with 50 mL per pot (10^7^ cells or conidia mL^–1^). PHC BioPak^®^ and T-Gro were dissolved in water and mixed with the soil three dbt to reach 10^7^ CFU g^–1^ of soil.

### Genome sequencing of SynCom 1.2 isolates

The genomes of *Pseudomonas* sp. PS5, *Bacillus* sp. BN8.2, and *Trichoderma* sp. T2C1.4, which then composed SynCom 1.2, were sequenced using the MinION platform (Oxford Nanopore Technologies, Oxford, United Kingdom). Overnight LB broth cultures (1 mL) of the bacteria or mycelium (100 mg) of T2C1.4 scraped off from 7-day-old cellophane-agar plates were used to extract the bacterial/fungal DNA using the Quick-DNA Fungal/Bacterial Miniprep kit (ZYMO Research, Irvine, CA, United States) following the manufacturer’s indications. The MIN-101B device was used with the flow cell R9.4.1 (FLO-MIN106D) and the Rapid Sequencing Kit (SQK-RAD004) according to the manufacturer’s instructions. One flow cell was used per sample, and the sequencing was run for 1 or 3 h to obtain 1 or 3 gigabases (Gb) for the bacteria and T2C1.4, respectively. The MinKnow software version 4.2.8 (Oxford Nanopore Technologies, Oxford, United Kingdom) was used for the sequencing run and basecalling using default parameters. Reads were *de novo* assembled using Canu version 2.2 with default parameters (Koren et al., 2017). The genome of the two bacterial isolates were annotated using the online version of the NCBI Prokaryotic Genome Annotation Pipeline (PGAP) version 6.1 (Li et al., 2020). The T2C1.4 genome was annotated using Augustus (Galaxy version 3.3.3) with default parameters (Stanke et al., 2008) within the Galaxy platform (version 20.01) (Afgan et al., 2016) and the result is reported in the Supplementary Material. The whole genome shotgun project has been deposited at DDBJ/ENA/GenBank under the BioProject ID PRJNA834929. The accession numbers of the three genomes and the sequencing reads are reported in Supplementary Table S2.

For the species identification, genomic sequences were extracted from the genomes and used in multilocus sequence analyses (MLSA) for *Pseudomonas* spp. (Gomila et al., 2015; Garrido-Sanz et al., 2016), *Bacillus* spp. (Niazi et al., 2014), and *Trichoderma* spp. (Kullnig-Gradinger et al., 2002). Multiple sequence alignments were performed using Clustal Omega (Sievers et al., 2011), and neighbor-joining phylogenetic trees (1000 bootstraps) using MEGA 11.0.10 (Tamura et al., 2021). The extracted sequences were deposited in GenBank under the accession numbers reported in Supplementary Table S2.

The presence of FA-resistance gene cluster (*fus*) was checked in the genomes of SynCom 1.2 isolates by BLAST analysis (blastn algorithm with *e*-value < 0.001) using *Burkholderia cepacia* (synonymous with *Pseudomonas cepacia*) FA-resistance operon as a query (GenBank accession number: D12503.1).

### Experimental design and statistical analysis

Statistical analyses were done with the software R ver. 4.1.1 (ISBN 3-900051-07-0^1^) within RStudio ver. 2021.09.0 build 351^2^. Plots were generated using the ggplot2 package ver. 3.3.5 (Wickham et al., 2016).

Categorical variables such as disease incidence, symptom severity, and vascular browning were analyzed by Friedman’s test, and treatments were compared by the Dunn’s test (*P* < 0.05). The abundance of microbial populations was log-transformed and subjected to the analysis of variance (ANOVA). The normality of distribution and homoscedasticity were previously ascertained using Shapiro–Wilk’s test and Bartlett’s test, respectively. The *t*-test (*P* < 0.05) was used to compare two samples (e.g., treated versus control), and the Tukey’s test (*P* < 0.05) for the multiple comparisons of the means.

Effective doses 50% (EC_50_) of FA were calculated using the DRC package in R. Before calculating EC_50_, the best model was fitted (e.g., Weibull, log-logistic, etc.) per each strain using the OD_595_ data of the last time point because they better described the sensitivity to FA. EC_50_ values, obtained per each replicate, were analyzed by ANOVA and the Tukey’s test (*P* < 0.05).

## Results

### Antagonists of *Fusarium oxysporum* f. sp. *cubense* were isolated from the banana rhizosphere

A total of 96 isolates were obtained from 35 banana rhizosphere samples: 23 *Pseudomonas* spp., 28 *Bacillus* spp., 16 *Streptomyces* spp., and 29 *Trichoderma* spp. (Figure 2 and Supplementary Table S1).

In dual culture assays, the maximum *Foc* TR4 inhibitions observed for *Pseudomonas* spp., *Bacillus* spp., and *Streptomyces* spp. were 63.1, 57.3, and 48.3%, respectively. Diffusible metabolites of *Trichoderma* spp., evaluated by the cellophane-agar method, were able to hinder the growth of *Foc* TR4 up to 39.9%. On the other hand, volatile compounds of these isolates, evaluated by the overlapping plate method, were not markedly toxic for the pathogen (maximum inhibition of 17%).

### SynCom 1.0 reduced Fusarium wilt severity

**FIGURE 3:**
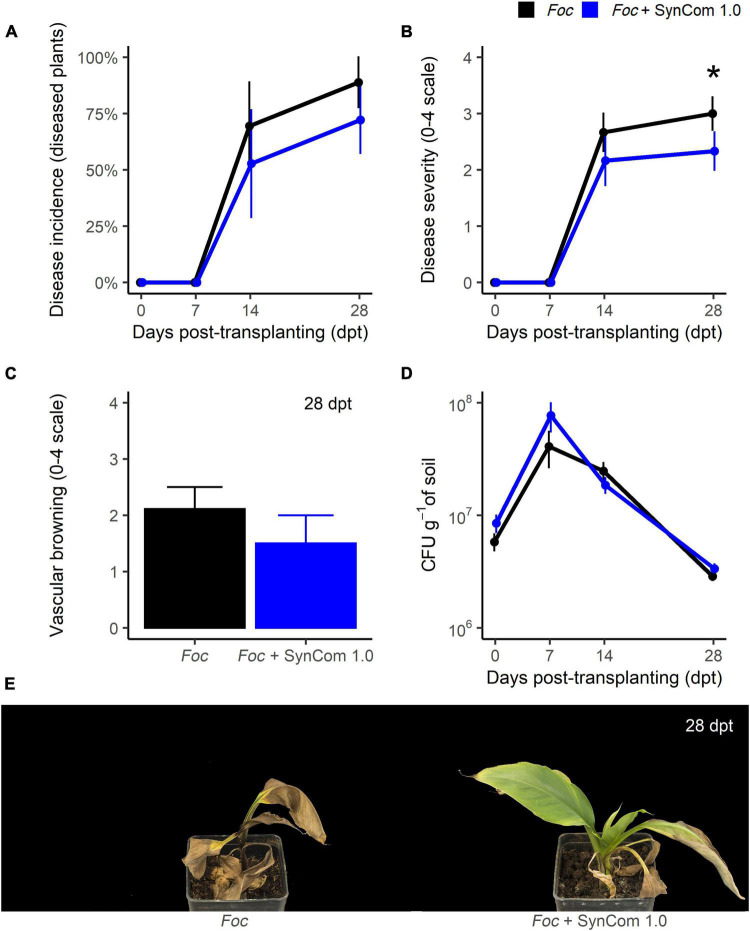
Biocontrol trial A: SynCom 1.0. Banana plants ‘Gran Enana’ were grown in steam-sterilized soil inoculated with 10^7^ CFU g^–1^ soil of *Fusarium oxysporum* f. sp. *cubense* (*Foc*) tropical race 4, alone or in combination with the synthetic microbial community SynCom 1.0. Fusarium wilt incidence **(A)**, symptom severity **(B)**, vascular browning **(C)**, and *Fusarium* spp. soil population **(D)** were measured to assess the biocontrol effectiveness of SynCom 1.0. Symptoms occurring on treated and control plants are shown in **(E)**. Bars indicate standard error (*n* = 15). The asterisk indicates a significant difference between treated and control plants according to Dunn’s test (*P* < 0.05).

In the biocontrol trial A, FWB symptoms appeared at 14 dpt in the control plants (Figure 3). Remarkably, SynCom 1.0 significantly (*P* < 0.05) reduced symptom severity by 35% at 28 dpt (Figures 3B,E), though no significant differences occurred in disease incidence (Figure 3A) and vascular discoloration (Figure 3C). SynCom 1.0 treatment significantly reduced *Fusarium* spp. abundance in the corm tissues at 28 dpt (Supplementary Figure S1D), viz. from 1.2 × 10^7^CFU g^–1^ in the control to 1.8 × 10^6^CFU g^–1^ in SynCom-treated plants. However, this reduction was not observed in the soil over time (Figure 3D). In fact, after inoculation with the pathogen at 10^7^ CFU g^–1^, *Foc* inoculum density in the soil raised to 10^8^ CFU g^–1^ at 7 dpt and then decreased (Figure 3D). This fluctuation over time was not observed in the soil inoculated with SynCom 1.0.

As expected, being leaf yellowing a typical FWB symptom, *Foc* infection caused a decrease in the leaf chlorophyll content in the control plants (Supplementary Figure S1A). In fact, while chlorophyll content in untreated control plants increased over time from 30 to 49.7 μg cm^–2^ because of the normal growth of the plants, it remained below 35 μg cm^–2^ in *Foc*-inoculated, untreated plants. SynCom 1.0 treatment did not significantly affect the chlorophyll content, regardless of whether or not plants were inoculated with *Foc* TR4. Populations of *Pseudomonas* spp., *Bacillus* spp., *Streptomyces* spp., and *Trichoderma* spp. remained at high levels during the entire experiment (Supplementary Figure S1B). Inoculated at 10^7^ CFU g^–1^ of soil, their population abundance increased at 7 dpt (or 14 dpt for *Pseudomonas* spp.) and decreased later on. Populations of the four genera remained above 10^6^ CFU g^–1^ of soil at the end of the experiment, and their fluctuations did not differ significantly over time except for *Bacillus* spp. (*P* < 0.0001). Inside the corm, these microorganisms were found at the end of the experiment: significant (*P* < 0.01) differences between SynCom-treated and -untreated plants were found for all the taxa except *Bacillus* spp. (Supplementary Figure S1C). Interestingly, *Trichoderma* spp. were only found in treated plants.

### The biocontrol effectiveness of SynCom 1.1 was similar to that of SynCom 1.0

**FIGURE 4:**
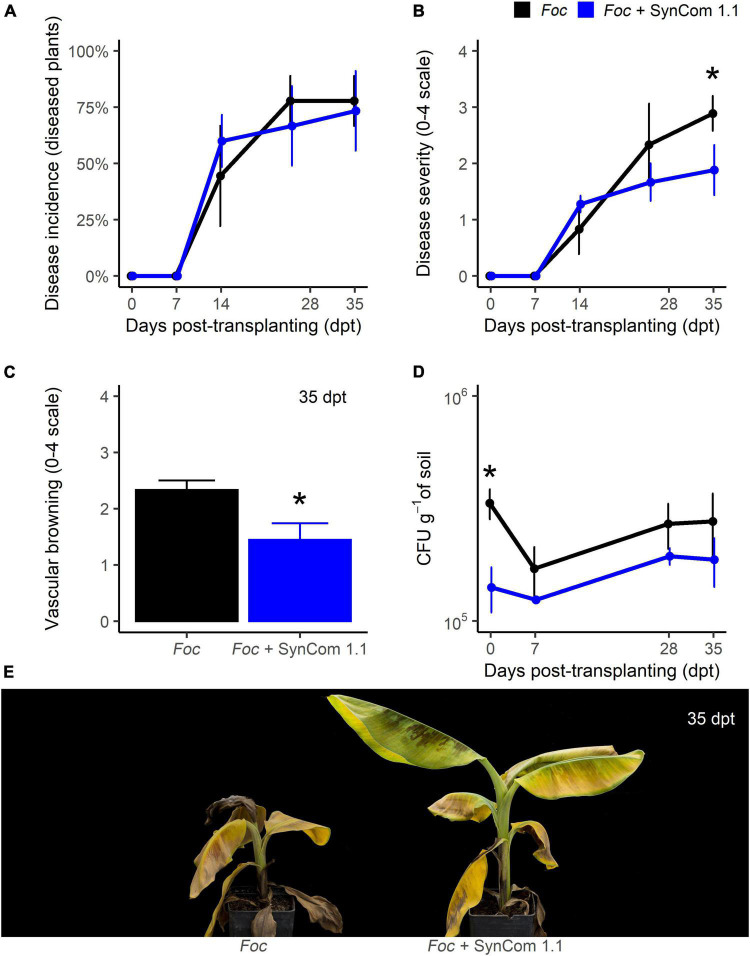
Biocontrol trial B: SynCom 1.1. Banana plants ‘Gran Enana’ were grown in steam-sterilized soil inoculated with 5.10^5^ CFU g^–1^ soil of *Fusarium oxysporum* f. sp. *cubense* (*Foc*) tropical race 4, alone or in combination with the synthetic microbial community SynCom 1.1. Fusarium wilt incidence **(A)**, symptom severity **(B)**, vascular browning **(C)**, and *Fusarium* spp. soil population **(D)** were measured to assess the biocontrol effectiveness of SynCom 1.0. Symptoms occurring on treated and control plants are shown in **(E)**. Bars indicate standard error (*n* = 3 in **A,D**, while *n* = 15 in **B,C**). Asterisks indicate significant differences between treated and control plants according to the Dunn’s test or the *t*-test (*P* < 0.05) in **(A–C,D)**, respectively.

In biocontrol trial B, the disease progress over time was very similar to the previous experiment, though it was conducted with less *Foc* inoculum (5.10^5^ CFU g^–1^ compared to 10^7^ CFU g^–1^ in biocontrol trial A) to reduce the high disease pressure observed in the previous experiment. Like in trial A, wilt symptoms appeared 14 dpt and no difference occurred in disease incidence over time (Figure 4A). At the end of the experiment, 35 dpt, symptom severity in SynCom-treated plants was significantly (*P* < 0.05) reduced by 35% compared to control (Figures 4B,E). Also, while in the control plants the vascular browning was 2.3 (on a 0–4 scale), in SynCom-treated plants it was 1.4 (*P* < 0.05; Figure 4C). At transplanting, *Foc* density in the soil was significantly lower in the SynCom-treated samples but no significant difference occurred later on (Figure 4D).

Another experiment with inoculated soil but without banana plants showed that the transient or null effect of SynCom 1.1 on *Foc* TR4 in the soil was not dependent on the initial pathogen inoculum density (10^3^, 10^5^, or 10^7^ CFU g^–1^) over 32 days (Supplementary Figure S2).

### Characterization of SynCom 1.1 isolates

#### SynCom 1.1 isolates possess several biocontrol traits

Isolates of SynCom 1.1 displayed several biocontrol and/or plant growth promotion-related traits (Table 1). Some of them were shared among isolates and genera while others showed specificity. For instance, *Bacillus* spp. were the sole isolates producing 2,3 butanediol, *Pseudomonas* spp. had phytase activity, while ACC deaminase activity was found only in *Streptomyces* sp. St2AOB1 and *Trichoderma* sp. T2C1.4. The three *Pseudomonas* spp. were almost identical based on the GEN III MicroPlate system, with a profile very similar to that of *Pseudomonas chlororaphis* (Supplementary Table S3).

**TABLE 1:** Traits related to biocontrol in SynCom 1.1 members and several other isolates used as controls.

Species	Strain	Activity of enzymes and production of substances as compared to control										

		ACC deaminase activity	2,3 butanediol production	Catalase activity	Siderophores production	Phytase activity	Phosphatase activity	β-glucosidase activity	Amylase activity	HCN production	Protease activity	NH_3_ production
***Isolates of this study***												
*Pseudomonas* spp.	PS5	–	–	+	+	±	–	–	–	+ +	+	+
	P1A1	–	–	±	+ +	±	–	–	–	+	+	+
	P1CI	–	–	+	++	±	–	–	–	+ +	+	+
*Bacillus* spp.	BN8.2	–	+ +	+	±	–	–	+ +	+ +	–	+ +	+ +
	BT1	n.d.	+	+	–	–	–	+	+	–	+ +	+
*Streptomyces* sp.	St2AOB1	±	–	++	n.d.	–	–	+	+	–	±	+
*Trichoderma* sp.	T2C1.4	+	–	+ +	n.d.	–	–	+	–	–	–	–
***Isolates used in our laboratory as references for these assays***												
*Pseudomonas* sp.	PICF6	+	n.d.	n.d.	n.d.	n.d.	n.d.	n.d.	n.d.	n.d.	n.d.	n.d.
*Pantoea agglomerans*	IAS-B-1036	n.d.	+	+	–	n.d.	n.d.	n.d.	n.d.	n.d.	n.d.	n.d.
*Pseudomonas simiae*	PICF7	–	–	+	+	+	+	–	–	–	+	n.d.
*Chryseobacterium* sp.	IAS-B-370	n.d.	n.d.	n.d.	n.d.	n.d.	n.d.	+	+	n.d.	+	n.d.
*P. chlororaphis*	IAS-B-1013	n.d.	n.d.	n.d.	n.d.	n.d.	n.d.	n.d.	n.d.	+	n.d.	n.d.
Unknown genus	IAS-B-717	n.d.	n.d.	–	n.d.	–	n.d.	n.d.	n.d.	n.d.	n.d.	n.d.
*Pseudomonas* sp.	PIC25	n.d.	n.d.	n.d.	n.d.	n.d.	–	n.d.	n.d.	n.d.	–	n.d.

The table is the result of two independent assays, each with two replicates (plate wells). ACC, 1-Aminocyclopropane-1-Carboxylate; HCN, cyanuric acid; NH_3_, ammonia; ++, strong activity/production; +, moderate activity/production; ±, borderline activity/production; –, no activity/production; n.d., not determined.

#### Beneficial microorganisms can also antagonize other beneficial microorganisms

Interactions amongst SynCom 1.1 isolates due to both their agar-diffusible metabolites and volatile compounds were studied. Not surprisingly, we observed that our beneficial microorganisms did not target only the pathogen, but other beneficial microbes as well.

**FIGURE 5:**
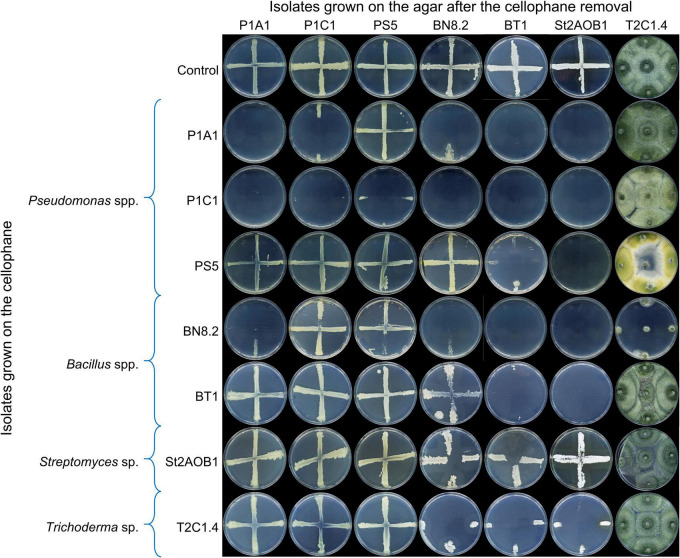
Interactions amongst SynCom 1.1 isolates: effect of metabolites released in the agar medium. Cellophane-agar method (potato dextrose agar). On the rows, isolates grown on the cellophane before its removal (viz. producing putatively toxic metabolites) are indicated. On the columns, isolates inoculated in the plates after cellophane removal (viz. isolates targeted by the metabolites) are indicated. Partial growth or null growth is indicative of sensitivity to metabolites released in the medium. Each plate is representative of four replicates.

The growth of the microorganisms on PDA containing microbial diffusible metabolites is shown in Figure 5. The upper row of plates shows the control: cellophane film was inoculated with water and, after its removal, the microorganisms were inoculated (e.g., P1A1, P1C1, etc.); the growth was optimal. The second row of plates, for example, shows the growth of the microorganisms (e.g., P1A1, P1C1, etc.) on PDA previously inoculated (on the cellophane disk) with *Pseudomonas* sp. P1A1. On these plates, *Pseudomonas* sp. PS5 and *Trichoderma* sp. T2C1.4 grew like in the control; therefore, they were resistant to metabolites produced by P1A1. In contrast, *Bacillus* sp. BT1 and *Streptomyces* sp. St2AOB1 did not grow at all, indicating they were sensitive to these metabolites. *Pseudomonas* sp. P1C1 and *Bacillus* sp. BN8.2 showed a faint growth (partially sensitive to the metabolites of P1A1). It is worth noting that *Pseudomonas* sp. P1A1 did not grow at all on this medium containing its metabolites, indicating self-toxicity. *Pseudomonas* sp. P1C1 (third row of plates in Figure 5) inhibited all tested bacteria (including itself) but not the fungus *Trichoderma* sp. T2C1.4. *Bacillus* sp. BT1 and *Streptomyces* sp. St2AOB1 (fifth and sixth column of plates) were sensitive to all microorganisms except St2AOB1. *Trichoderma* sp. T2C1.4 (seventh column of plates) was resistant to all microorganisms except *Pseudomonas* sp. PS5 and *Bacillus* sp. BN8.2, which induced severe stress (yellow pigmentation instead of green) or complete growth inhibition, respectively, in T2C1.4.

The growth of the microorganisms exposed to microbial volatile compounds is shown in Supplementary Figure S3. Overall, there were no remarkable effects of volatile compounds. Nevertheless, *Trichoderma* sp. T2C1.4 showed some stress (no pigmentation and/or reduced growth) when exposed to *Pseudomonas* spp. and *Bacillus* spp. volatiles. Finally, volatiles emitted by *Bacillus* sp. BT1 completely inhibited this fungus.

#### *Fusarium oxysporum* f. sp. *cubense* defends itself by antagonizing beneficial microorganisms and fusaric acid is involved in such antagonism

**FIGURE 6:**
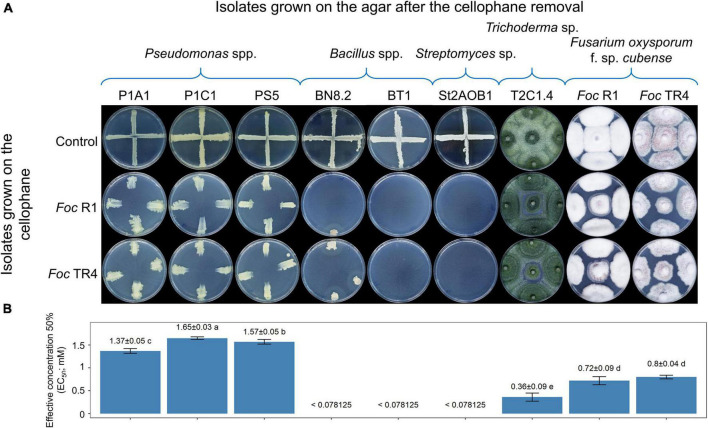
Effect of *Fusarium oxysporum* f. sp. *cubense* (*Foc*) on the growth of SynCom 1.1 isolates. Effect of *Foc* race 1 (R1) or tropical race 4 (TR4) metabolites in the cellophane-agar method **(A)** and determination of the effective doses of commercial fusaric acid **(B)**. In **(A)**, on the rows, isolates grown on the cellophane before its removal (viz. producing putatively toxic metabolites) are indicated. On the columns, isolates inoculated in the plates after cellophane removal (viz. isolates targeted by the metabolites) are indicated. Partial growth or null growth is indicative of sensitivity to metabolites released in the medium. Each plate is representative of four replicates. In **(B)**, bars indicate standard error (*n* = 3). Means with different letters are significantly different according to Tukey’s test (*P* < 0.05).

The antagonistic activity among beneficial microorganisms stimulated us to investigate whether they can be targeted by *Foc* as well. Figure 6A shows the effects of the metabolites released by *Foc* R1 and TR4 into the PDA against the seven isolates of SynCom 1.1. Surprisingly, metabolites of the two *Foc* races had some effects even against themselves; indeed, *Foc* colonies grown on PDA containing their metabolites were slightly smaller than the control, indicating some self-toxic effect. The same limited (not null, though) effect was observed for *Trichoderma* sp. T2C1.4. Three *Pseudomonas* spp. isolates were inhibited only in the center of the plates, viz. where the highest concentration of metabolites occurred (i.e., the inoculation point of the *Foc* agar plug). The growth of *Bacillus* sp. BN8.2 was drastically reduced, whereas *Bacillus* sp. BT1 and *Streptomyces* sp. St2AOB1 did not grow at all, indicating the high sensitivity of these isolates to *Foc* metabolites.

Since FA is a well-known pathogenicity factor of *F. oxysporum* isolates, including *Foc*, we hypothesized an important role of this compound in the interaction with other microorganisms. Interestingly enough, EC_50_ data (Figure 6B) correlated well with the Petri plate assay (Figure 6A). In fact, EC_50_ values for BN8.2, BT1, and St2AOB1 were below the lowest concentration tested, i.e., 0.078125 mM, indicating high sensitivity to FA. In contrast, *Foc* strains and *Trichoderma* sp. T2C1.4 were resistant (EC_50_ values of 0.6, 0.72, and 0.8 mM, respectively). Remarkably, according to this assay, the three *Pseudomonas* spp. isolates were even more resistant than *Foc*, as they showed higher EC_50_ values (ranging between 1.37 and 1.65 mM). Further details on the EC_50_ calculation, including fitting to the models, are reported in Supplementary Figure S4.

### The optimized consortium: SynCom 1.2

Information on the dual interactions gathered from the previous experiments (summarized in Supplementary Figure S5A) was taken into account for the most optimal application of the components of this consortium. SynCom 1.2 was constructed with the most antagonistic and cross-compatible isolates: *Pseudomonas* sp. PS5, *Bacillus* sp. BN8.2 and *Trichoderma* sp. T2C1.4. *Pseudomonas* spp. P1A1 and P1C1 were discarded because they antagonized most of the beneficial microorganisms (Figure 5). *Bacillus* sp. BT1 and *Streptomyces* sp. St2AOB1 were also removed because they were strongly inhibited by *Foc* (Figure 5).

**FIGURE 7:**
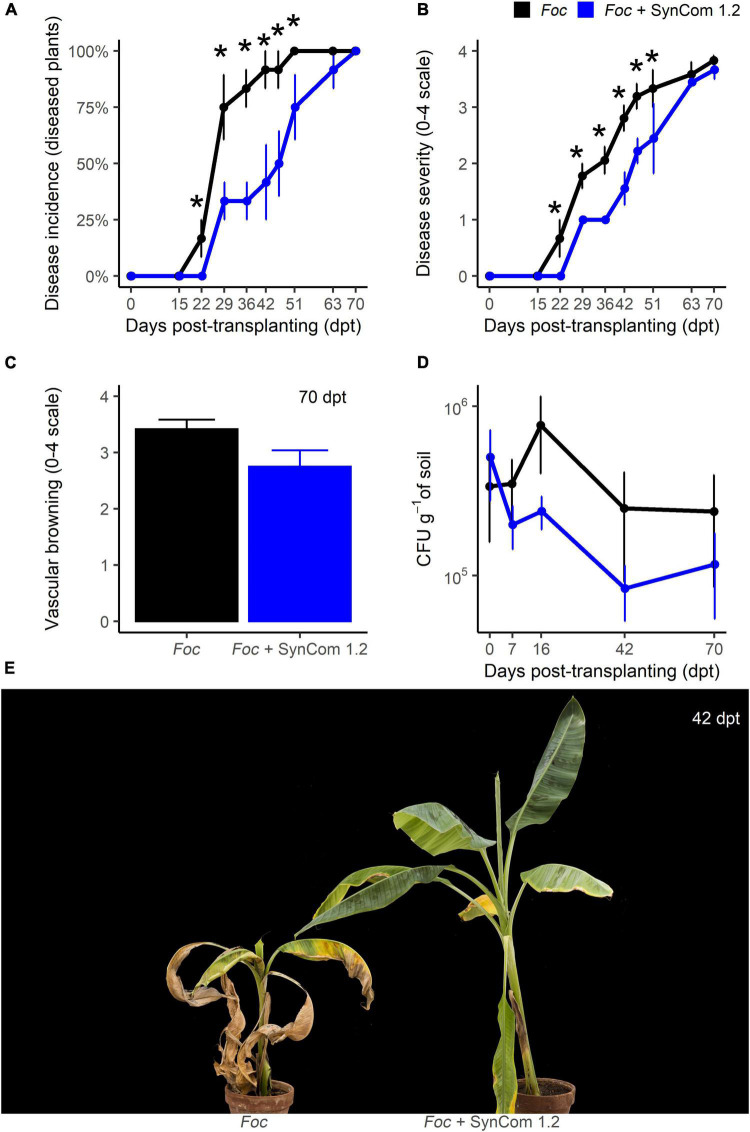
Biocontrol trial C: SynCom 1.2. Banana plants ‘Gran Enana’ were grown in steam-sterilized soil inoculated with 5.10^5^ CFU g^–1^ soil of *Fusarium oxysporum* f. sp. *cubense* tropical race 4, alone or in combination with the synthetic microbial community SynCom 1.2. Fusarium wilt incidence **(A)**, symptom severity **(B)**, vascular browning **(C)**, and *Fusarium* spp. soil population **(D)** were measured to assess the biocontrol effectiveness of SynCom 1.2. Symptoms occurring on treated and control plants are shown in **(E)**. Bars indicate standard error (*n* = 3 in **A,D**, while *n* = 15 in **B,C**). Asterisks indicate significant differences between treated and control plants according to Dunn’s test (*P* < 0.05).

A new protocol for the inoculation of SynCom 1.2 was then designed (Supplementary Figure S5B). Using this protocol and a *Foc* density of 5.10^5^ CFU g^–1^ of soil, FWB symptoms appeared at 22 dpt in control plants of experiment C, the highest disease incidence (100%) occurred at 51 dpt, and the highest symptom severity (4 on a 0–4 scale) was reached at 70 dpt. Disease incidence was significantly (*P* < 0.05) reduced between 22 and 51 dpt in SynCom-treated plants compared to control, while such a difference disappeared later on and until the end of the experiment, 70 dpt (Figure 7A). An identical pattern was observed when scoring the symptom severity (Figures 7B,E). For example, at 42 dpt, incidence and severity were reduced by 55 and 44%, respectively. No significant differences occurred in vascular browning and *Foc* density in the soil (Figures 7C,D). Therefore, in this experiment, SynCom 1.2 provided better results than those obtained with SynCom 1.1 in trial B (both experiments were carried out with 5.10^5^ CFU of *Foc* TR4 per gram of soil).

**FIGURE 8:**
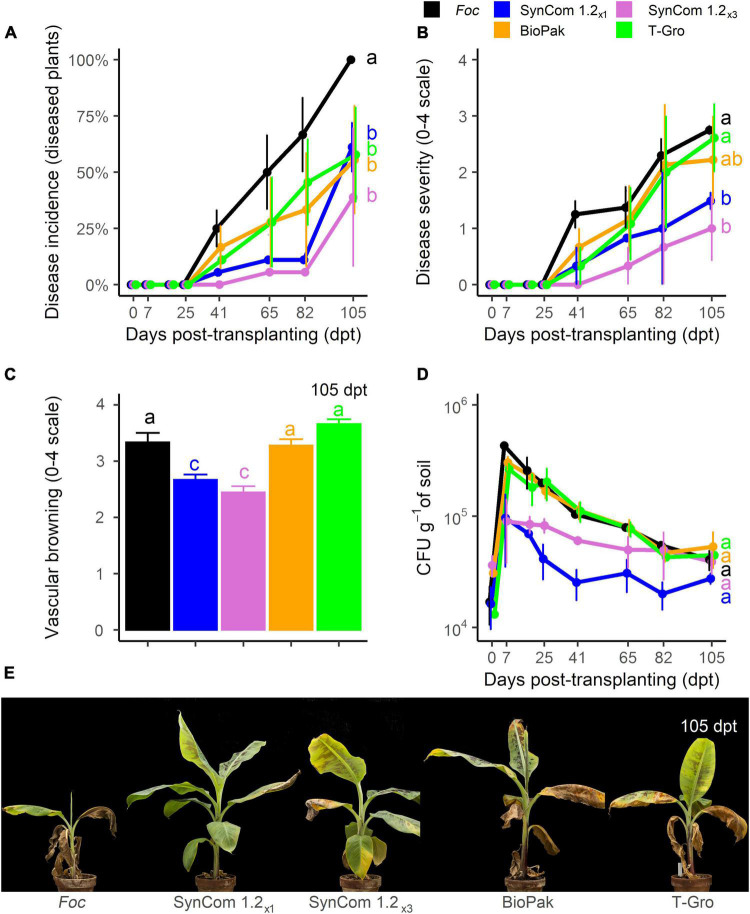
Biocontrol trial D: SynCom 1.2 and commercial products. Banana plants ‘Gran Enana’ were grown in steam-sterilized soil inoculated with 10^4^ CFU g^–1^ soil of *Fusarium oxysporum* f. sp. *cubense* (*Foc*) tropical race 4, alone or in combination with diverse biological control agents, either experimental (i.e., SynCom 1.2, composed of three isolates, applied once, i.e., SynCom 1.2_×1_, or three times, i.e., SynCom 1.2_×3_) or commercial (i.e., BioPak and T-Gro). Fusarium wilt incidence **(A)**, symptom severity **(B)**, vascular browning **(C)**, and *Fusarium* spp. soil population **(D)** were measured to assess the biocontrol effectiveness of SynCom 1.2. Symptoms occurring on treated and control plants are shown in **(E)**. Bars indicate standard error (*n* = 3 in **A,D**, while *n* = 15 in **B,C**). Means with different letters are significantly different according to Dunn’s test (*P* < 0.05; in the line plots, means comparisons are shown only at the last time point for better readability of the graph).

In the biocontrol trial D (10^4^ CFU g^–1^ of *Foc* TR4), control plants showed FWB symptoms starting from 41 dpt, disease incidence reached 100% at 105 dpt, the end of the experiment (Figure 8A), but symptom severity never reached the maximum value (Figure 8B). All the treatments significantly (*P* < 0.05) reduced disease incidence at the end of the experiment (up to 62% reduction observed for SynCom 1.2_×3_) compared to control, while no difference occurred among them (Figure 8A). Interestingly, SynCom 1.2_×1_ and SynCom 1.2_×3_ treatments were equally effective in reducing symptom severity (average scores of 1.49 and 1, respectively, compared to 2.75 in the control) while symptoms on BioPak^®^- (2.2) and T-Gro-treated plants (2.6) were not significantly different from the control (Figures 8B,E). Moreover, a significant reduction in vascular browning was observed in all treatments but T-Gro (Figure 8C). While vascular browning severity reached a value of 3.3 in the control, lower values were scored for SynCom 1.2_×1_ (2.7) and SynCom 1.2_×3_ (2.4) treatments. As in the previous experiments, we observed an increase in soil *Foc* population at 7 dpt (4.3.10^5^ CFU g^–1^, compared to 1.7.10^4^ CFU g^–1^ at transplanting), and a slight decrease later on (Figure 8D). Such an increase at 7 dpt was hindered in soil samples treated with SynCom 1.2_×1_ or SynCom 1.2_×3_ (9.7.10^5^ and 9.10^5^ CFU g^–1^, respectively). At the end of the experiment, no significant difference was detected among the treatments, including the control. Therefore, it can be concluded that SynCom 1.2 was more effective than the commercially available bioproducts BioPak^®^ and T-Gro.

### Genome sequences and species identification of SynCom 1.2 isolates

The complete genome sequence was obtained for *Pseudomonas* sp. PS5 and *Bacillus* sp. BN8.2, while a near-complete sequence was obtained for *Trichoderma* sp. T2C1.4. The statistics of genome sequencing and assembling are reported in Supplementary Table S4.

For the isolate PS5, a run of 2 h and 22 min yielded 241,360 sequences (raw reads) for a total of 1.09 gigabases (Gb; Supplementary Table S4). After correction and trimming, 6.9% of reads (25.8% of bases) were retained and used for the assembling, which successfully generated one circular chromosome (i.e., the complete genome) of 7,107,906 bp (7.1 Mb) with 62.7% GC content. With these reads, the genome coverage was 40×. For the isolate BN8.2, a run of 2 h and 55 min generated 374,370 reads for a total of 1.22 Gb (Supplementary Table S4). Corrected, trimmed reads (4.8%) were assembled into one circular chromosome (i.e., the complete genome) of 4,030,079 bp (4 Mb) with 46.5% GC content. The genome was covered 43×. For the isolate T2C1.4, a run of 12 h produced 894,230 reads for a total of 2.58 Gb (Supplementary Table S4). The assembling of corrected, trimmed reads (25.4%) yielded 10 contigs of the nuclear genome (40.8 Mb, 47.4% GC content) and one circular DNA for the mitochondrion (57,176 bp, 27.8% GC content). Overall, the coverage was 39×.

A summary of the genome annotations is reported in Supplementary Table S5. A total of 6,597, 4,150, and 14,703 genes were identified in PS5, BN8.2, and T2C1.4, respectively. In the bacterial genomes, a high number of pseudogenes were identified (17 and 27% for PS5 and BN8.2, respectively).

The MLSA allowed us to identify the species of the SynCom isolates. In the phylogenetic tree of *Pseudomonas* spp., PS5 clustered with 100% bootstraps (1000) with isolates of *P. chlororaphis* subsp. *piscium* (Supplementary Figure S6), which formed a clade distinct from other subspecies. Therefore, PS5 was identified as *P. chlororaphis* subsp. *piscium*. BN8.2 was identified as *B. velezensis* because it clustered (98% bootstraps) with the type strain (FZB42) of *B. amyloliquefaciens* subsp. *plantarum* (synonym with *B. velezensis*) (Supplementary Figure S7). T2C1.4 was identified as *T. virens* because it clustered (98% bootstraps) with three isolates of this species (Supplementary Figure S8).

## Discussion

For a long time, biological control has gained great attention from both scientists and farmers because of its environmental sustainability. On the other hand, it has suffered from inconstant effectiveness over time and across environments. In the last decades, mixtures of microorganisms, either antagonists or plant growth promoters, microbial consortia, synthetic microbial communities, and/or *ad hoc* tailored microbiomes have surfaced to increase and maintain the effectiveness of BCAs (Gopal et al., 2013; Mercado-Blanco et al., 2018).

Sometimes, effective microbial antagonists of plant pathogens have been searched in disease-suppressive soils, where they should be more abundant (Shen et al., 2015). However, even if established in a disease-conducive soil, the rhizosphere is a good reservoir of plant beneficial microbes (Gómez-Lama Cabanás et al., 2018a,b). We successfully isolated *Foc*-antagonists from the banana rhizosphere of farms located on Tenerife Island with no history of disease suppressiveness. *In vitro* growth inhibition of *Foc* mycelium by selected antagonists reached 60% although complete growth suppression was not observed for any of them (Figure 2). This was not a surprising outcome since different *formae speciales* of *F. oxysporum* have been earlier reported as resistant to microbial antagonists in similar *in vitro* studies. For instance, *Foc* R1 and TR4 showed resistance to volatile compounds emitted by *Pseudomonas protegens* CHA0, while several Basidiomycetes and Ascomycetes were sensitive (Prigigallo et al., 2021). Also, *F. oxysporum* f. sp. *lycopersici* was among the less sensitive soil-borne fungi in an *in vitro* screening for antagonism of 27 *Streptomyces* spp. isolates (Bubici, 2006).

SynCom 1.0 was constructed with 44 isolates with the rationale to obtain a consortium of microorganisms exerting moderate/high antagonism levels against *Foc* TR4. Due to its size, such a community should be expected to efficiently compete with the target pathogen. Our idea was to mimic a natural microbiota manipulated by agricultural practices to favor its beneficial portion (hence not only highly effective antagonists) and suppress plant pathogens (Orozco-Mosqueda et al., 2018; Compant et al., 2019; De Corato, 2020, 2021). A second synthetic microbial community, SynCom 1.1, was constructed with the most effective isolates (seven). Results showed that both SynComs yielded similar control of FWB. In both cases, symptom severity was significantly reduced (by 35%) but disease incidence (percentage of disease plants) was not, very likely because a higher number of plants were necessary for a statistical significance of percentage data (Figures 3, 4). Therefore, the hypothesis that a large community of *Foc*-antagonists could cope with *Foc* better than a small community was not confirmed. Moreover, in these experiments and another one performed without plants, the SynComs were not able to reduce *Foc* inoculum in the soil (Figures 3, 4 and Supplementary Figure S2). Hence, the reduction of symptom severity could be the consequence of a diminished ability of *Foc* to penetrate the roots, colonize the plant tissues, or just induce the symptom expression, but not the consequence of killing *Foc* in the soil (Köhl et al., 2019). Yet, we cannot exclude that SynComs, or some of their constituents, may induce defense responses in bananas, as already demonstrated for other microbial consortia (Akila et al., 2011; Sarma et al., 2015).

To improve the effectiveness of the SynCom, the interactions among SynCom 1.1 isolates and between them and *Foc* TR4 were investigated. The study of the interactions among microorganisms is one of the principles for the design of a SynCom (Smith, 2011; Johns et al., 2016; de Souza et al., 2020). We discovered an undesired, though expected, phenomenon: beneficial microorganisms not only antagonized *Foc*, but also antagonized among them in some cases (including themselves), and *Foc* antagonized beneficial microorganisms as well. While the self-inhibition of microorganisms (e.g., P1A1, P1C1, etc.) could be interpreted as a mechanism of population’s growth regulation mediated by their metabolites (Hayes and Low, 2009), the antagonism among different beneficial microorganisms would be largely unsought. For instance, *Pseudomonas* sp. P1C1 inhibited all the other beneficial bacteria and *Streptomyces* sp. St2AOB1 was sensitive to all the beneficial microorganisms (cellophane-agar assay; Figure 2). This kind of side effect of beneficial microorganisms has not been reported often in the literature (Gómez-Lama Cabanás et al., 2018b; Izquierdo-García et al., 2020). One possible explanation is that negative results in this type of studies are likely discarded or not considered worthy of publishing. The concept that BCAs can also exert undesired effects has been emerging recently. Like pathogens, beneficial microbes can sometimes suppress or evade the plant immune system, which in turn shapes microbiome assembly in response to environmental variations (Teixeira et al., 2019). In our opinion, these (and probably other) undesired effects might contribute to exemplifying more complex microbial networks, and could explain the inconstant results of BCAs (Gómez-Lama Cabanás et al., 2022). Indeed, should an effective antagonist be hampered by another one in a two-way interaction *in vitro*, it is reasonable to think about its difficulty to cope with a soil microbiota populated with thousands of diverse species either beneficial, neutral, or pathogenic for the plants. Ideally, a well-structured SynCom would be more stable in the environment and more resilient to disturbing factors like environmental conditions or indigenous microbiota, because the role or function of a given member could be partially covered by another one (Czajkowski et al., 2020).

We also discovered that *Foc* exerted a potent antagonistic activity against the beneficial microbes here selected. Moreover, we observed that FA, *viz*. one of the metabolites produced by *Fusarium* species (Liu et al., 2020), might be involved in such an antagonistic activity (Figure 3). However, the production of FA by the *Foc* strain used in this research would need a further demonstration. FA has been known to participate in the infection process of banana plants by *Foc* TR4 (Liu et al., 2020) and the amount produced by the mycelium has correlated with the symptom severity induced on the plant (Li et al., 2013). We showed that FA is not only a pathogenicity factor (Liu et al., 2020) but also a tool used by this pathogen in microbe-microbe interactions to defend itself, protect its niche, and outcompete in the environment. *Pseudomonas* spp. were the most resistant isolates to a commercial FA (EC_50_ = 1.4–1.7 mM), even more than *Foc* (EC_50_ = 0.7–0.8 mM; self-toxicity), while *Bacillus* spp. and *Streptomyces* sp. were the most sensitive ones (EC_50_ < 0.078 mM). Previous research already demonstrated that *P. fluorescens* strain Pf10 could grow in a medium containing 100 μg mL^–1^ FA (= 0.56 mM) and could even detoxify this toxin (Thangavelu et al., 2001). The FA-resistance gene cluster (*fus*) was first discovered in *B. cepacia* (Utsumi et al., 1991) and is present in the genome of several bacteria, but no information is available for fungal genomes (according to our search in the NCBI nucleotide database). In the genome of our *Pseudomonas* sp. PS5, we found a region with a 66% sequence similarity (77% coverage) with the *B. cepacia fus* gene cluster, but no homologous region was found in *Bacillus* sp. BN8.2 and *Trichoderma* sp. T2C1.4. We observed a good tolerance of *Trichoderma* sp. T2C1.4 to FA (EC_50_ = 0.36 mM). It would be interesting to evaluate whether the resistance to FA or the detoxification capability of microorganisms could be used as a new method for screening BCA candidates.

With this knowledge, we designed the SynCom 1.2 with only three isolates: *Pseudomonas* sp. PS5, *Bacillus* sp. BN8.2, and *Trichoderma* sp. T2C1.4. However, since some *in vitro* antagonism occurred among these isolates, we opted for a non-simultaneous application in the biocontrol trial C: after inoculation of *Foc* into the soil, PS5 and T2C1.4 were inoculated into the soil six and three dbt and BN8.2 was inoculated by root-dipping at transplanting (Supplementary Figure S5). Using this strategy, a significant improvement in biocontrol effectiveness was obtained compared to the two previous SynComs here designed. In this trial, the disease progress over time was significantly delayed, though no difference from the control occurred at the end of the experiment (Figure 6). In the next trial (D), a significant reduction in symptom severity in SynCom 1.2-treated plants was observed at the end of the experiment (Figure 7). The timing of BCA applications along with the type of formulation has been reported to affect significantly the biocontrol level (Raguchander et al., 2000; Akila et al., 2011). Kavino and Manoranjitham (2018) proposed the *in vitro* bacterization of banana plantlets with endophytes and rhizobacteria for the successive control of FWB.

To understand whether we achieved promising biocontrol, SynCom 1.2 was compared to two commercial BCAs (trial D). Mukhongo et al. (2015) have reported that BioPak^®^ and Eco-T^®^ (according to the manufacturer, the latter product is a synonym with T-Gro, which was used in our trial) decreased soil *Foc* population with a magnitude that was soil type-dependent, though no biocontrol trials were performed. This did not occur in our trial where, however, SynCom 1.2 slightly decreased the *Foc* population during the early stages of the experiment but not at its end (Figure 7D). Also, plants treated with SynCom 1.2 showed the severity of foliar symptoms and vascular browning significantly lower than the control and plants treated with BioPak^®^ and T-Gro (Figures 7B,C). This confirmed the good potential of SynCom 1.2, which would merit further testing in field trials.

Finally, we showed that the minION platform can be a useful, cheap, and rapid technique to sequence the genome of BCAs and hence to gain knowledge on their repertoire of biocontrol traits as well as accurate species identification. Either for *Pseudomonas* sp. PS5 or *Bacillus* sp. BN8.2, sequencing runs of less than 3 h yielded over 1 Gb each, which was enough to assemble the complete genomes. In these genomes, a high number of pseudogenes were identified. Pseudogenes are genes with the coding sequence without complete protein, as predicted upon homology with proteins in the databases. An in-depth inspection of the sequences of these pseudogenes (e.g., BLAST analysis) revealed the occurrence of single-nucleotide deletions in homopolymers (e.g., AAAAA, TTTTTT, etc.). These deletions caused frameshifts in the reading frame and thus internal stop codons and truncated predicted proteins. This is well-known trouble affecting the Oxford Nanopore Technologies platforms (Delahaye and Nicolas, 2021) and computational tools developed so far (Loman et al., 2015; Sarkozy et al., 2018; Arumugam et al., 2019) cannot solve completely the problem. In our study, more pseudogenes were detected in BN8.2, which genome is poorer in guanine-thymine (GC) content (46.5%) compared to PS5 (62.7%). This is not in agreement with Delahaye and Nicolas (2021), who demonstrated that low-GC reads have fewer errors than high-GC reads. Hence, very likely factors other than GC content affected the accuracy of sequencing and assembling of our genomes.

For *Trichoderma* sp. T2C1.4, a 12 h-run yielded 2.68 Gb and a near-complete genome was obtained: the mitochondrion sequence and 10 contigs of the nuclear genome. The near-completeness of this genome represents a significant advance in the genomics of the *Trichoderma* genus. The sole publicly available genome of *T. virens* published so far (the strain Gv29-8) is constituted of 94 contigs (Kubicek et al., 2011). In the *T. harzianum* species, for instance, only one (strain CGMCC 20739) out of nine genomes has been resolved at the chromosome level (BioProject PRJNA695870). That genome is composed of seven chromosomes, suggesting a very low fragmentation level of the T2C1.4 assembly (10 contigs). For our isolate, at least four chromosomes could be considered complete (telomere-to-telomere) because of the presence of predicted centromere (AT-reach large region; Seidl et al., 2020) and telomeres (TTAGGG repeats; Wu et al., 2009). The other chromosomes were probably fragmented in the remaining six contigs.

The availability of the genome sequence of BCAs is a valuable resource for gaining new knowledge about them. For example, from the genomes of SynCom 1.2 isolates, we retrieved sequences useful for molecular species identification, avoiding synthesis of oligonucleotides, polymerase chain reactions (PCR), cloning (or purification), and sequencing of many PCR products. Moreover, with the genome sequenced, we were able to ascertain the presence of the FA-resistance gene cluster.

## Conclusion

Designing a synthetic microbial community is not an easy task. The mere mixtures of antagonists (e.g., SynCom 1.0 and 1.1) might provide effective biocontrol, but an accurate investigation of the interactions among beneficial microorganisms is needed to obtain superior results (e.g., SynCom 1.2). Microorganisms, either beneficial or pathogenic, adopt several mechanisms to compete in the environment. By showing undesired effects such as the antagonism of *Foc* against beneficial microorganisms, we highlighted a phenomenon sometimes neglected in the literature, though extremely reasonable: it is hard to believe that a beneficial microorganism acts only against plant pathogens and, *vice versa*, that a plant pathogen is affected by beneficial microorganisms without any attempt of defense.

We present an example of the workflow for constructing a SynCom for the biological control of plant pathogens, including a characterization of microorganisms spanning from the detection of biocontrol traits to the sequencing of the genome. Finally, novel BCAs effective against *Foc* TR4 were identified.

## Data availability statement

The datasets presented in this study can be found in online repositories. The names of the repository/repositories and accession number(s) can be found in the article/Supplementary Table S2.

## Author contributions

GB and MIP conceived the research, isolated the microorganisms, performed the *in vitro* and *in planta* experiments, and wrote the manuscript. CG-LC and JM-B sampled the soil and supervised the physiological characterization of the microorganisms. All the authors revised the manuscript.

## Footnotes

1
http://www.Rproject.org

2
http://www.rstudio.com
