# Supplementary material for: Designing a synthetic microbial community devoted to biological control: The case study of Fusarium wilt of banana
Source: Front Microbiol. 2022 Aug 5;13:967885. doi: 10.3389/fmicb.2022.967885 (PMC9389584; doi:10.3389/fmicb.2022.967885)
Supplement: Supplementary file 2 [file Data_Sheet_2.zip › Table 1.DOCX]

Table S1. Locations (in Tenerife Island, Spain) of the soil samples used to isolate beneficial microorganisms.

| **Locality*^a^*** | **Latitude, Longitude** | **Microbial isolates obtained in this research*^b^*** |
| --- | --- | --- |
| F01 | 28°10'06" N, 16°26'13" W | P: **P1A1**, P1B1, **P1C1**, P1D1; B: B1A1, B1B1, B1E2; S: St1A1, St1D1 |
| F02 | 28°09'00" N, 16°47'36" W | P: P2B1, P2C1.2.1, P2C1.2.2, P2C1.4.1, P2D1, P2E1; B: B2A1, B2B1, B2C1.2, B2C1.2.1; S: St2A1.2, St2B1, St2C1.1, St2C1.2; T: T2A1.1, T2A1.2, T2B1.1, T2B1.2, T2C1.1, T2C1.2, T2C1.3, **T2C1.4**, T2E1 |
| F03 | 28°11'02" N, 16°47'01" W | P: P3B1, P3E1; B: B3A1, B3A1.1, B3A1.2, B3B1, B3C1, B3E1; S: St3A1, St3B1.1, St3B1.2, St3C1, St3D1, St3E1; T: T3A1.2 |
| F04 | 28°22'36" N, 16°44'08" | P: P4A, P4B1, P4D1.4.2; B: B4A1, B4B1.1, B4B1.2, B4C1, B4D1.4.1, B4D1.4.2; S: St4C1 |
| F09 | 28°29'46" N, 16°25'15" W | P: P4AOD1, P5AOE1; B: B2AOB1, B2AOD1, B4OD1; S: **St2AOB1**, St3AOC1, St4AOD1 |
| N2 | 28°22'00.1" N, 16°48'14.6" W | T: TN2 |
| N5 | 28°22'01.8" N, 16°48'25.2" W | T: TN5.1, TN5.2, TN5.3 |
| N7 | 28°22'37.0" N, 16°44'07.0" W | T: TN7.1, TN7.2, TN7.3 |
| N8 |  | B: BN8.1, **BN8.2** |
| S1 | 28°10'07.4" N, 16°26'14.6" W | B: **BT1**; T: TS1 |
| S3 |  | P: PS3.1 |
| S4 | 28°09'20.3" N, 16°48'00.6" W | T: TS4.1, TS4.2 |
| S5 |  | P: **PS5**, PSF5, PSFg, PSFy; B: BS5; T: TSFw, TSFy |
| S6 |  | P: PS6; B: BS6; T: TS6 |
| S7*^c^* | 28°12'25.5" N, 16°49'37.2" W | B: BS7; T: TS7 |
| S8*^c^* |  | T: TS8 |
| S9*^c^* |  | T: TS9.1, TS9.2, TS9.3, TS9.4 |

*^a^* Further information on F01, F02, F03, F04, and F09 localities are reported in Gómez-Lama Cabanás et al. (2021); from each of those localities, five soil samples (A to E) were taken. Information on the other samples (N2 to N8 and S1 to S9) is reported in Colagiero et al. (2021).

*^b^* P = *Pseudomonas* spp.; B = *Bacillus* spp.; S = *Streptomyces* spp.; T = *Trichoderma* spp. Isolates in bold were included in SynCom 1.1 (7), and those in bold underlined in SynCom 1.2 (3). The 44 isolates included in SynCom 1.0 were not highlighted here to avoid confusion (they are indicated in Figure 1).

*^c^* S7, S8, and S9 fields were planted with banana variety Gruesa, while the other locations with variety Pequeña Enana (synonymous with Dwarf Cavendish).
