# Supplementary material for: Designing a synthetic microbial community devoted to biological control: The case study of Fusarium wilt of banana
Source: Front Microbiol. 2022 Aug 5;13:967885. doi: 10.3389/fmicb.2022.967885 (PMC9389584; doi:10.3389/fmicb.2022.967885)
Supplement: Supplementary file 2 [file Data_Sheet_2.zip › Table 2.DOCX]

Table S2. Accession numbers of the genomes, sequencing reads, and genomic sequences used in the multilocus sequence analyses of the SynCom 1.2 isolates.

| **SynCom 1.2 isolates** | **Genome**  (BioProject ID PRJNA834929) | | |  | **Multilocus sequence analysis** | |
| --- | --- | --- | --- | --- | --- | --- |
|  | **BioSamlpe ID** | **Assembly**  **(accession number)** | **FASTQ sequences**  **(accession number)** |  | **Gene** | **Accession**  **number** |
| *Pseudomonas* sp. PS5 | SAMN28084625 | CP097271 | SRX15248154 |  | *16S ribosomal RNA* (*rRNA*) | ON394883 |
|  |  |  |  |  | *DNA gyrase subunit beta* (*gyrB*) | ON492011 |
|  |  |  |  |  | *DNA-directed RNA polymerase subunit beta* (*rpoB*) | ON492012 |
|  |  |  |  |  | *RNA polymerase sigma factor* (*rpoD*) | ON492013 |
| *Bacillus* sp. BN8.2 | SAMN28084626 | CP097288 | SRX15248155 |  | *DNA gyrase subunit A* (*gyrA*) | ON492017 |
|  |  |  |  |  | *DNA polymerase III subunit tau* (*dnaX*) | ON492018 |
|  |  |  |  |  | *Serine hydroxymethyltransferase* (*glyA*) | ON492019 |
|  |  |  |  |  | *Cysteine-tRNA ligase* (*cysS*) | ON492020 |
|  |  |  |  |  | *Glycerol uptake facilitator protein* (*glpF*) | ON492021 |
|  |  |  |  |  | *Guanylate kinase* (*gmK*) | ON492022 |
|  |  |  |  |  | *DNA gyrase subunit beta* (*gyrB*) | ON492023 |
|  |  |  |  |  | *DNA ligase A* (*ligA*) | ON492024 |
|  |  |  |  |  | *DNA repair protein* (*recN*) | ON492025 |
| *Trichoderma* sp. T2C1.4 | SAMN28084627 | JAMBQI000000000 | SRX15248156 |  | *Ribosomal RNA* (*rRNA*)*, internal transcribed spacers* (*ITS*) | ON399101 |
|  |  |  |  |  | *Mitochondrial small subunit* (*mitSSU*) | ON492014 |
|  |  |  |  |  | *Translation elongation factor 1-alpha* (*tef1*) | ON492015 |
|  |  |  |  |  | *Endochitinase 42* (*ech42*) | ON492016 |
