# Supplementary material for: Designing a synthetic microbial community devoted to biological control: The case study of Fusarium wilt of banana
Source: Front Microbiol. 2022 Aug 5;13:967885. doi: 10.3389/fmicb.2022.967885 (PMC9389584; doi:10.3389/fmicb.2022.967885)
Supplement: Supplementary file 2 [file Data_Sheet_2.zip › Table 3.DOCX]

Table S3. Result of the assays conducted with the GEN III MicroPlate™ (Biolog, Hayward, CA, USA) on three *Pseudomonas* spp. isolates composing SynCom 1.1.

| **Carbon sources** | ***Pseudomonas* spp. isolates** | | |  | **Carbon sources** | ***Pseudomonas* spp. isolates** | | |
| --- | --- | --- | --- | --- | --- | --- | --- | --- |
|  | **PS5** | **P1A1** | **P1C1** |  |  | **PS5** | **P1A1** | **P1C1** |
| Dextrin | - | - | - |  | D-Galacturonic Acid | - | - | - |
| D-Maltose | - | - | - |  | L-Galactonic Acid Lactone | - | - | - |
| D-Trehalose | + | + | + |  | D-Gluconic Acid | + | + | + |
| D-Cellobiose | - | - | - |  | D-Glucuronic Acid | - | - | - |
| Gentiobiose | - | - | - |  | Glucuronamide | +/- | - | - |
| Sucrose | + | - | - |  | Mucic Acid | + | + | + |
| D-Turanose | - | - | - |  | Quinic Acid | + | + | + |
| Stachyose | - | - | - |  | D-Saccharic Acid | + | + | + |
| D-Raffinose | - | - | - |  | p-Hydroxy-Phenylacetic Acid | + | + | + |
| α-D-Lactose | - | - | - |  | Methyl Pyruvate | - | - | - |
| D-Melibiose | - | - | - |  | D-Lactic Acid Methyl Ester | - | - | - |
| β-Methyl-DGlucoside | - | - | - |  | L-Lactic Acid | + | + | + |
| D-Salicin | - | - | - |  | Citric Acid | + | + | + |
| N-Acetyl-DGlucosamine | +/- | + | + |  | α-Keto-Glutaric Acid | +/- | + | + |
| N-Acetyl-β-DMannosamine | - | - | - |  | D-Malic Acid | +/- | - | - |
| N-Acetyl-DGalactosamine | - | - | - |  | L-Malic Acid | + | + | + |
| N-Acetyl Neuraminic Acid | - | - | - |  | Bromo-Succinic Acid | - | - | +/- |
| α-D-Glucose | + | + | + |  | Tween 40 | - | - | - |
| D-Mannose | +/- | +/- | +/- |  | γ-Amino-Butyric Acid | + | + | + |
| D-Fructose | +/- | +/- | - |  | α-Hydroxy-Butyric Acid | - | - | - |
| D-Galactose | - | - | - |  | β-Hydroxy-D,L butyric Acid | + | + | +/- |
| 3-Methyl Glucose | - | - | - |  | α-Keto-Butyric Acid | - | - | - |
| D-Fucose | +/- | - | - |  | Acetoacetic Acid | - | - | - |
| L-Fucose | - | - | - |  | Propionic Acid | + | + | + |
| L-Rhamnose | - | - | - |  | Acetic Acid | + | + | + |
| Inosine | - | - | - |  | Formic Acid | - | - | - |
| D-Sorbitol | - | - | - |  |  |  |  |  |
| D-Mannitol | +/- | +/- | +/- |  | **Chemicals** |  |  |  |
| D-Arabitol | - | +/- | - |  | pH 6 | + | + | + |
| Myo-inositol | +/- | +/- | +/- |  | pH 5 | + | + | + |
| Glycerol | +/- | +/- | +/- |  | 1% NaCl | + | + | + |
| D-Glucose-6-PO4 | - | - | - |  | 4% NaCl | + | + | + |
| D-Fructose-6-PO4 | - | - | - |  | 8% NaCl | - | - | - |
| D-Aspartic Acid | - | - | - |  | 1% sodium lactate | + | + | + |
| D-Serine | + | + | + |  | Fusidic acid | + | + | + |
| Gelatin | - | - | - |  | D-serine | + | + | + |
| Glycyl-L-Proline | +/- | +/- | - |  | Troleandomycin | + | + | + |
| L-Alanine | +/- | + | +/- |  | Rifamycin SV | + | + | + |
| L-Arginine | + | + | +/- |  | Minocycline | + | + | + |
| L-Aspartic Acid | + | + | + |  | Lincomycin | + | + | + |
| L-Glutamic Acid | + | + | + |  | Guanidine HCl | + | + | + |
| L-Histidine | + | + | + |  | Niaproof 4 | + | + | + |
| L-Pyroglutamic Acid | + | + | + |  | Vancomycin | + | + | + |
| L-Serine | + | + | + |  | Tetrazolium violet | + | + | + |
| Pectin | - | - | - |  | Tetrazolium blue | + | + | + |
|  |  |  |  |  | Nalidixic acid | + | + | + |
|  |  |  |  |  | Lithium chloride | - | - | - |
|  |  |  |  |  | Potassium tellurite | + | + | + |
|  |  |  |  |  | Aztreonam | + | + | + |
|  |  |  |  |  | Sodium butyrate | - | - | - |
|  |  |  |  |  | Sodium bromate | - | - | - |

+= positive; -= negative; ±= borderline.
