# Supplementary material for: Designing a synthetic microbial community devoted to biological control: The case study of Fusarium wilt of banana
Source: Front Microbiol. 2022 Aug 5;13:967885. doi: 10.3389/fmicb.2022.967885 (PMC9389584; doi:10.3389/fmicb.2022.967885)
Supplement: Supplementary file 2 [file Data_Sheet_2.zip › Table 4.DOCX]

Table S4. Statistics of the genome sequencing and assembling of the three SynCom 1.2 isolates.

|  | **Raw reads** | **Basecalled reads**  **(q-score=7)*^a^*** | **Corrected reads** | **Corrected trimmed reads** | **Assembly** | |
| --- | --- | --- | --- | --- | --- | --- |
|  |  |  |  |  | **Genomic DNA** | **Mitochondrion DNA** |
| ***Pseudomonas* sp. PS5** Run duration: 2 h 22 m |  |  |  |  |  |  |
| baseparis (bp) | 1,090,000,000 | 1,026,215,933 | 282,254,648 | 280,835,688 | 7,107,906 |  |
| Percentage of the raw reads basepairs |  | 94.1% | 25.9% | 25.8% |  |  |
| Number of sequences | 241,360 | 218,081 | 16,543 | 16,542 | 1 |  |
| Percentage of the raw reads |  | 90.4% | 6.9% | 6.9% |  |  |
| GC content |  | 62.5% | 62.9% | 62.9% | 62.7% |  |
| length N50 |  | 8,328 | 16,839 | 16,765 | 7,107,906 |  |
| Maximum length |  | 91,266 | 50,060 | 49,991 | 7,107,906 |  |
| Mean length |  | 4,705 | 17,061 | 16,977 | 7,107,906 |  |
| Median length |  | 3,086 | 15,849 | 15,787 | 7,107,906 |  |
| Minimum length |  | 1 | 1,018 | 1,018 | 7,107,906 |  |
| Coverage | 153 | 144 | 40 | 40 |  |  |
|  |  |  |  |  |  |  |
| ***Bacillus* sp. BN8.2** Run duration: 2 h 55 m |  |  |  |  |  |  |
| baseparis (bp) | 1,220,000,000 | 1,140,602,526 | 173,855,009 | 172,275,035 | 4,030,079 |  |
| Percentage of the raw reads basepairs |  | 93.5% | 14.3% | 14.1% |  |  |
| Number of sequences | 374,370 | 333,782 | 17,858 | 17,796 | 1 |  |
| Percentage of the raw reads |  | 89.2% | 4.8% | 4.8% |  |  |
| GC content |  | 46.7% | 46.7% | 46.7% | 46.5% |  |
| length N50 |  | 6,044 | 13,719 | 13,652 | 4,030,079 |  |
| Maximum length |  | 45,889 | 42,728 | 39,037 | 4,030,079 |  |
| Mean length |  | 3,417 | 9,735 | 9,680 | 4,030,079 |  |
| Median length |  | 2,177 | 11,758 | 11,724 | 4,030,079 |  |
| Minimum length |  | 1 | 1,000 | 1,000 | 4,030,079 |  |
| Coverage | 303 | 283 | 43 | 43 |  |  |
|  |  |  |  |  |  |  |
| ***Trichoderma* sp. T2C1.4** Run duration: 12 h |  |  |  |  |  |  |
| baseparis (bp) | 2,580,000,000 | 2,514,818,582 | 1,609,307,008 | 1,595,813,281 | 40,784,612 | 57,176 |
| Percentage of the raw reads basepairs |  | 97.5% | 62.4% | 61.9% |  |  |
| Number of sequences | 894,230 | 826,316 | 219,888 | 219,862 | 10 | 1 |
| Percentage of the raw reads |  | 92.4% | 24.6% | 24.6% |  |  |
| GC content |  | 46.8% | 47.6% | 47.7% | 47.4% | 27.8% |
| length N50 | 5,340 | 5,612 | 7,769 | 7,718 | 5,310,918 | 57,176 |
| Maximum length |  | 45,456 | 36,471 | 36,463 | 8,105,832 | 57,176 |
| Mean length |  | 3,043 | 7,318 | 7,258 | 4,078,461 | 57,176 |
| Median length |  | 1,836 | 6,443 | 6,391 | 4,534,721 | 57,176 |
| Minimum length |  | 1 | 1,071 | 1,008 | 36,501 | 57,176 |
| Coverage | 63 | 62 | 39 | 39 |  |  |

*^a^* Basecalling was done using MinKnow software, while correction, trimming, and assembling of the reads were done using Canu version 2.2.
