# Supplementary material for: Designing a synthetic microbial community devoted to biological control: The case study of Fusarium wilt of banana
Source: Front Microbiol. 2022 Aug 5;13:967885. doi: 10.3389/fmicb.2022.967885 (PMC9389584; doi:10.3389/fmicb.2022.967885)
Supplement: Supplementary file 2 [file Data_Sheet_2.zip › Table 5.DOCX]

Table S5. Statistics of the genome annotation of the three SynCom 1.2 isolates.

| **Annotation*^a^*** | **SynCom 1.2 isolates** | | |
| --- | --- | --- | --- |
|  | ***Pseudomonas* sp. PS5** | ***Bacillus* sp. BN8.2** | ***Trichoderma* sp. T2C1.4** |
| Genes (total) | 6,597 | 4,098 | 14,703 |
| CDSs (total) | 6,510 | 3,980 | 14,703 |
| CDSs with protein | 5,377 | 2,882 |  |
| Pseudogenes (total; CDSs without protein) | 1,133 | 1,098 |  |
| Pseudogenes: frameshifted | 1,048 | 1,030 |  |
| Pseudogenes: incomplete | 120 | 98 |  |
| Pseudogenes: internal stop | 19 | 12 |  |
| Pseudogenes: multiple problems | 52 | 38 |  |
| Genes (RNA) | 87 | 118 |  |
| rRNAs | 6, 5, 5  (5S, 16S, 23S) | 9, 9, 9  (5S, 16S, 23S) |  |
| tRNAs | 67 | 86 |  |
| ncRNAs | 4 | 5 |  |

*^a^* The genomes of *Pseudomonas* sp. PS5 and *Bacillus* sp. BN8.2 were annotated using the NCBI Prokaryotic Genome Annotation Pipeline (PGAP), and that of *Trichoderma* sp. T2C1.4 using Augustus.
