# Supplementary material for: Designing a synthetic microbial community devoted to biological control: The case study of Fusarium wilt of banana
Source: Front Microbiol. 2022 Aug 5;13:967885. doi: 10.3389/fmicb.2022.967885 (PMC9389584; doi:10.3389/fmicb.2022.967885)
Supplement: Supplementary file 3 [file Data_Sheet_3.zip › Figure S1.DOCX]

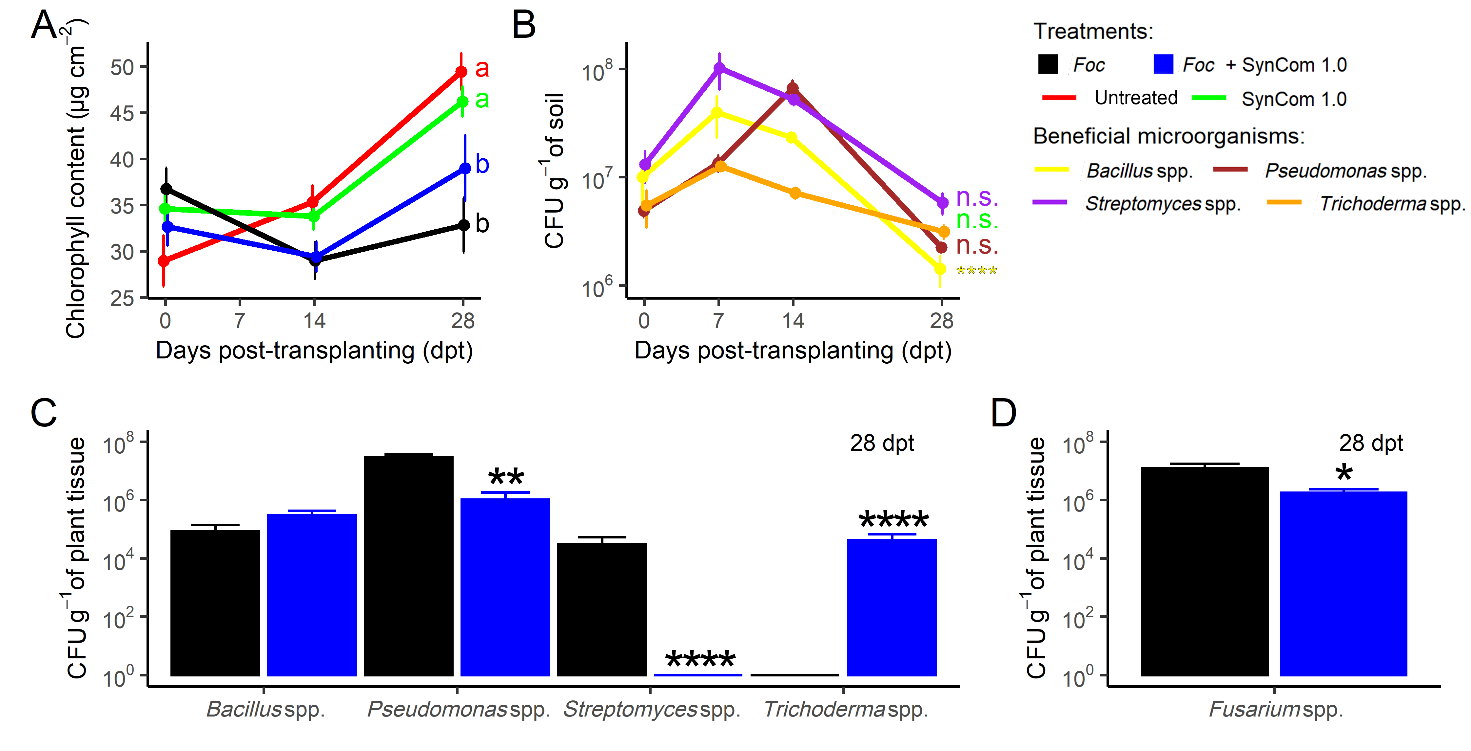


Figure S1. Biocontrol trial A: banana plants ‘Gran Enana’ inoculated with *Fusarium oxysporum* f. sp. *cubense* (*Foc*) tropical race 4 (TR4) and SynCom 1.0. Leaf chlorophyll content (A), population dynamics of the beneficial microorganisms (B), their abundance in the corm (C), and *Fusarium* spp. colonization of the xylem tissue (D). Bars indicate standard error (n=15 in A, n=3 in B, C, and D). Means with different letters are significantly different according to Tukey’s test (*P*<0.05; n.s.=not significant; in the line plots, means comparisons are shown only at the last time point for better readability of the graph). In B, asterisks indicate significant differences between the first and last time points according to the t-test (****= *P*<0.0001; n.s.=not significant, i.e., *P*≥0.05). In C and D, asterisks indicate significant differences between treated and control samples according to the t-test (*= *P*<0.05; **= *P*<0.01; ****= *P*<0.0001; n.s.=not significant, i.e., *P*≥0.05).
