# Supplementary material for: Designing a synthetic microbial community devoted to biological control: The case study of Fusarium wilt of banana
Source: Front Microbiol. 2022 Aug 5;13:967885. doi: 10.3389/fmicb.2022.967885 (PMC9389584; doi:10.3389/fmicb.2022.967885)
Supplement: Supplementary file 3 [file Data_Sheet_3.zip › Figure S2.DOCX]

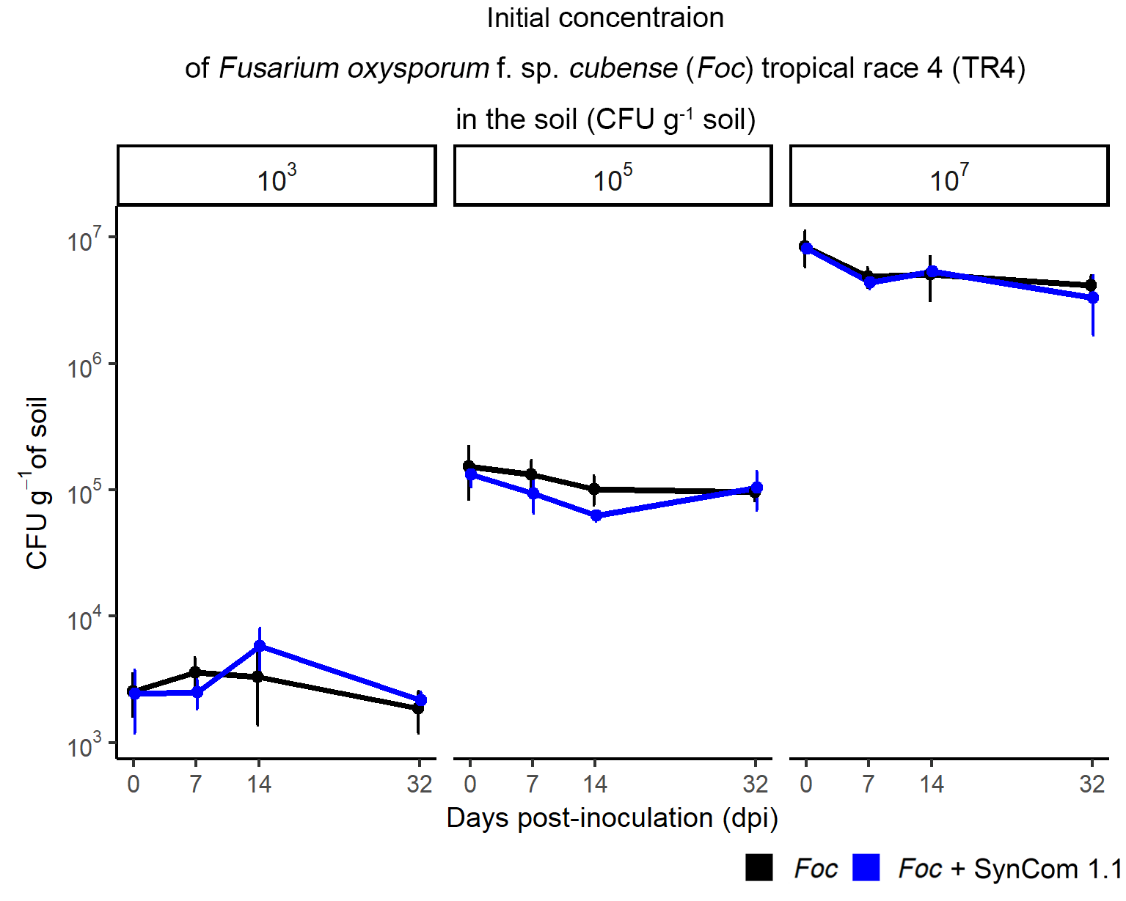


**Figure S2.** Population dynamics of *Fusarium* spp. in steam-sterilized soil artificially inoculated with *Fusarium oxysporum* f. sp. *cubense* (*Foc*) tropical race 4 (TR4) at several concentrations (10^3^, 10^5,^ or 10^7^ CFU g^-1­^ of soil) with or without the synthetic microbial community SynCom 1.1. Data were obtained from the dilution plate technique with the Komada’s medium starting from 5 g of soil (see Materials and Methods). Error bars indicate the standard error of the mean (n=4). No significant reduction of *Foc* was detected according to the t-test (P≥0.05).
