# Supplementary material for: Designing a synthetic microbial community devoted to biological control: The case study of Fusarium wilt of banana
Source: Front Microbiol. 2022 Aug 5;13:967885. doi: 10.3389/fmicb.2022.967885 (PMC9389584; doi:10.3389/fmicb.2022.967885)
Supplement: Supplementary file 3 [file Data_Sheet_3.zip › Figure S3.DOCX]

**
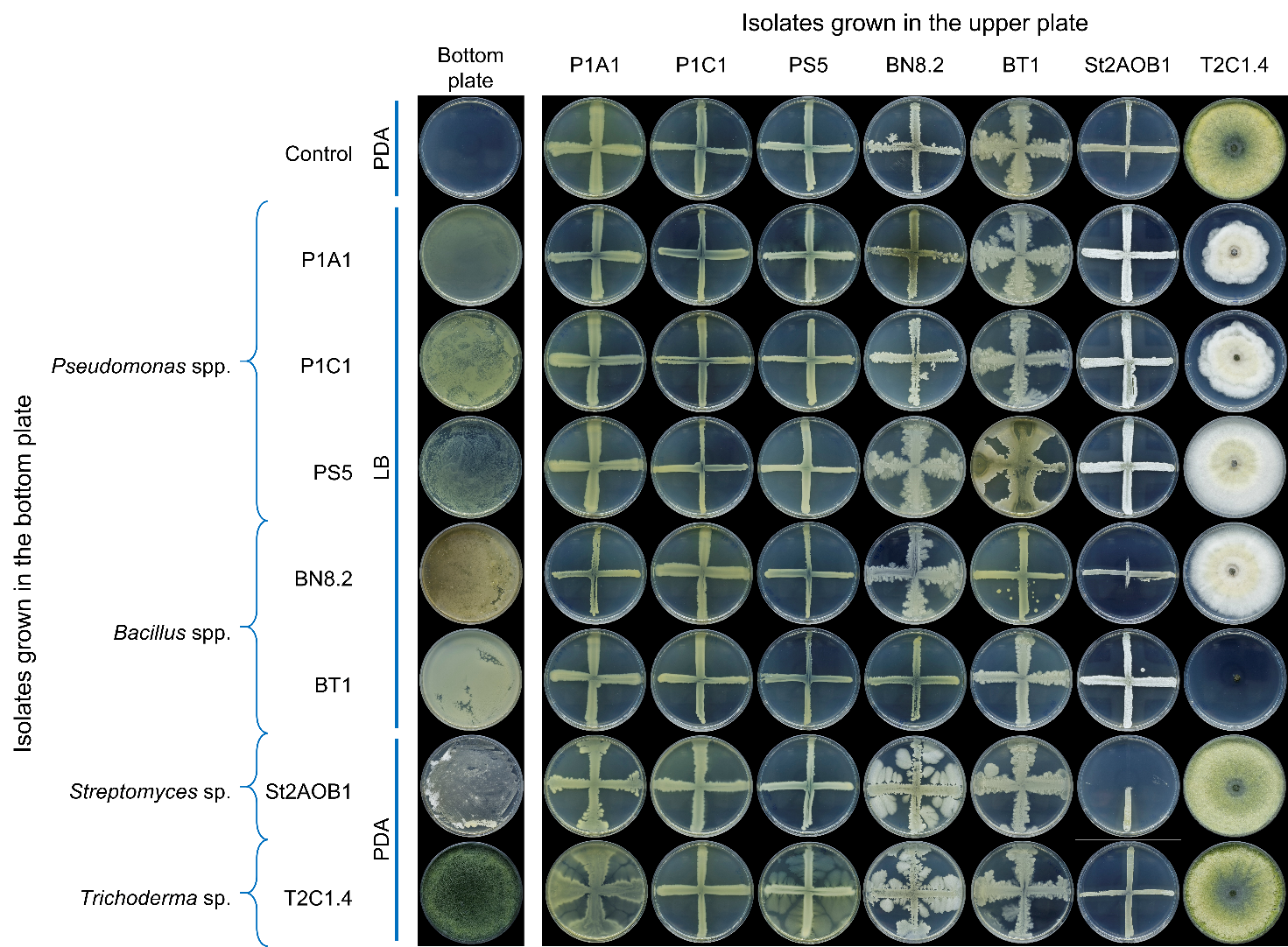
**

Figure S3. Interactions amongst SynCom 1.1 isolates: effect of volatile compounds. Overlapping plate method (potato dextrose agar or PDA, or Luria-Bertani agar or LB). On the rows, isolates grown in the bottom plate are reported; they are the isolates emitting putatively toxic volatile compounds. On the columns, isolates grown in the upper plate are reported; they are the target of volatile compounds. Partial growth or null growth is indicative of sensitivity to volatile compounds. Each plate is representative of four replicates.
