# Supplementary material for: Designing a synthetic microbial community devoted to biological control: The case study of Fusarium wilt of banana
Source: Front Microbiol. 2022 Aug 5;13:967885. doi: 10.3389/fmicb.2022.967885 (PMC9389584; doi:10.3389/fmicb.2022.967885)
Supplement: Supplementary file 3 [file Data_Sheet_3.zip › Figure S4.DOCX]

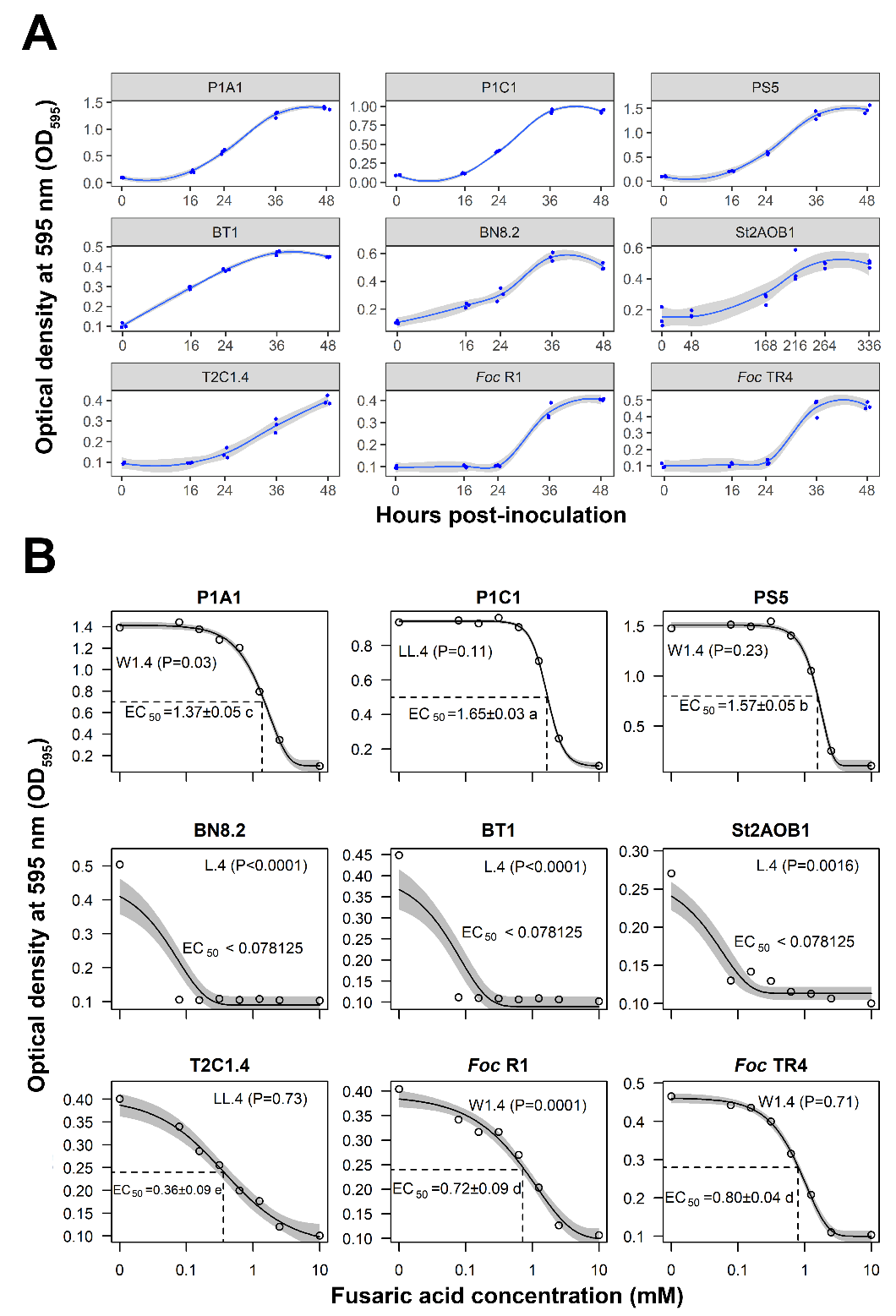


Figure S4. Sensitivity of SynCom 1.1 isolates and *Fusarium oxysporum* f. sp. *cubense,* both race 1 (*Foc* R1) and tropical race 4 (*Foc* TR4) to fusaric acid. Growth curves of potato dextrose broth cultures alone (A) or amended with fusaric acid at concentrations between 10 and 0.078125 mM (B) (n = 4). The shadow around the curves is the 95% confidence interval. In B, the best fitting model (e.g., W1.4 or Weibull 1.4, LL4 or log-logistic 4, etc.), corresponding *P*-value, and effective concentration 50% (EC_50_) are reported.
