# Supplementary material for: Designing a synthetic microbial community devoted to biological control: The case study of Fusarium wilt of banana
Source: Front Microbiol. 2022 Aug 5;13:967885. doi: 10.3389/fmicb.2022.967885 (PMC9389584; doi:10.3389/fmicb.2022.967885)
Supplement: Supplementary file 3 [file Data_Sheet_3.zip › Figure S5.DOCX]

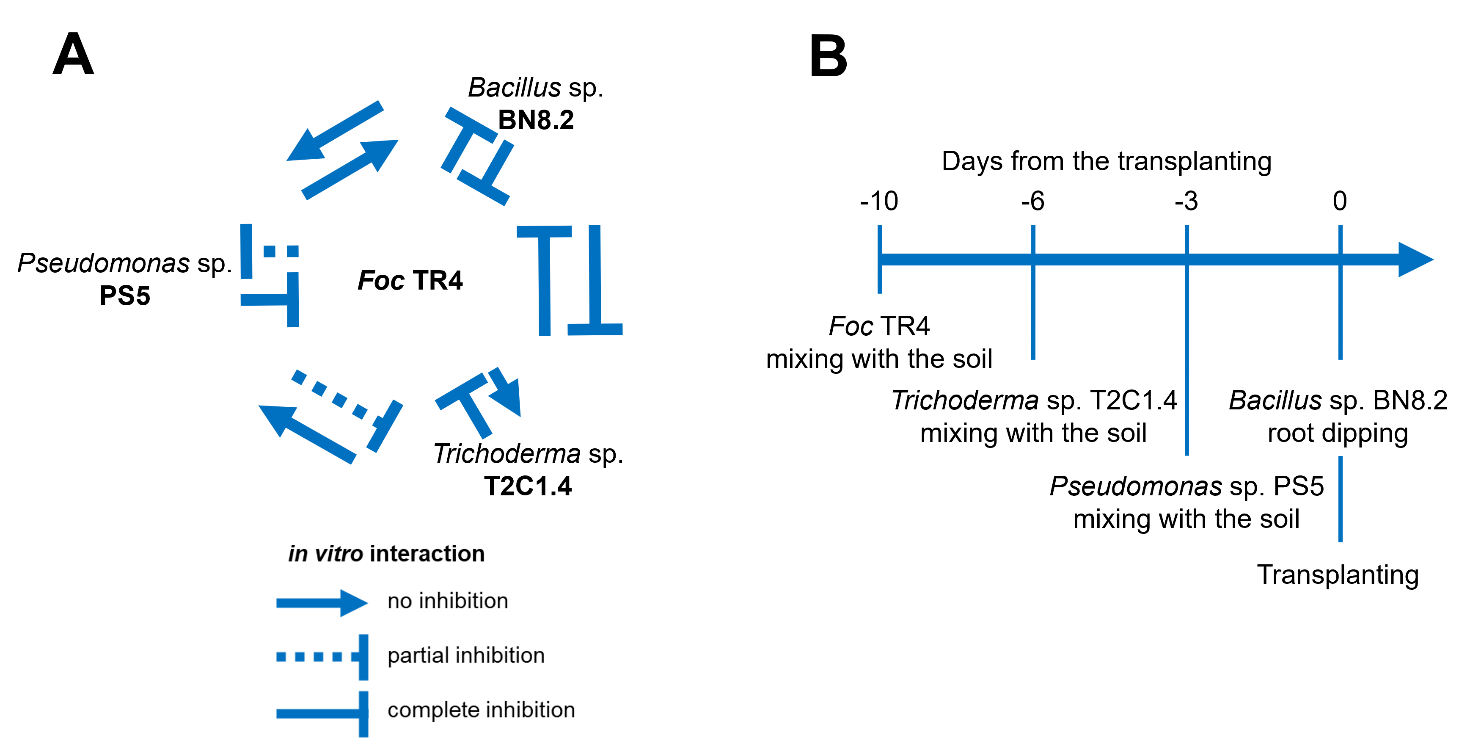


Figure S5. Principles of the SynCom 1.2 design and its application. Schematic representation of the interactions among SynCom 1.2 isolates (A) which led to the choice of three isolates to be included in this community, and the timing of their application in the biocontrol trials (B).
