# Supplementary material for: Designing a synthetic microbial community devoted to biological control: The case study of Fusarium wilt of banana
Source: Front Microbiol. 2022 Aug 5;13:967885. doi: 10.3389/fmicb.2022.967885 (PMC9389584; doi:10.3389/fmicb.2022.967885)
Supplement: Supplementary file 3 [file Data_Sheet_3.zip › Figure S6.DOCX]

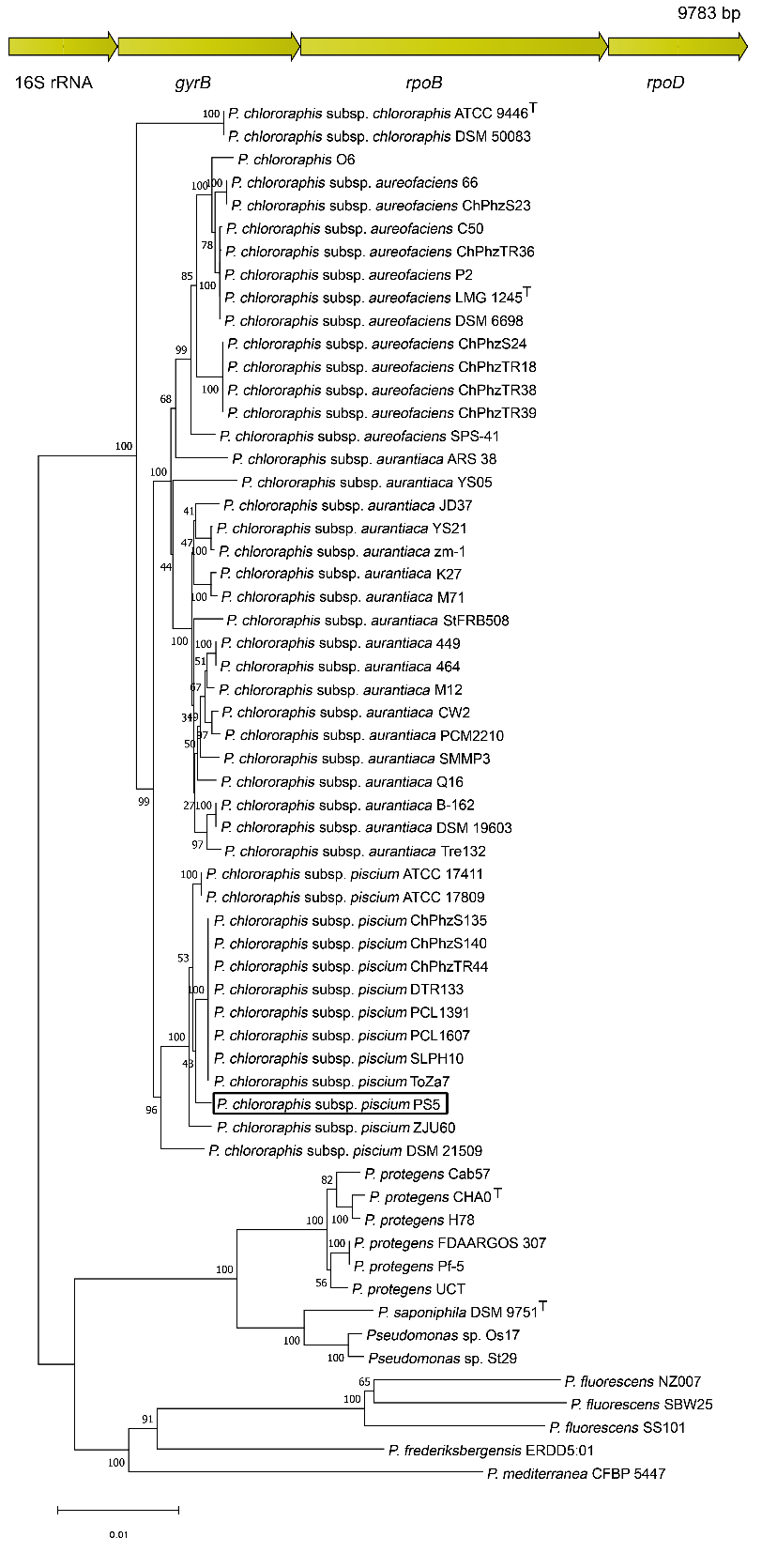


Figure S6. Neighbor-joining phylogenetic tree of 61 *Pseudomonas* spp. isolates constructed with the concatenated sequences of four genes (16S rRNA, *DNA* *gyrase subunit B* or *gyrB*, *DNA-directed RNA polymerase subunit beta* or *rpoB*, and *rpoD*). Percentage of 1000 bootstraps are reported on the branch nodes. The bar below the tree represents the branch distance scale. Type strains are labeled with T.
