# Supplementary material for: Designing a synthetic microbial community devoted to biological control: The case study of Fusarium wilt of banana
Source: Front Microbiol. 2022 Aug 5;13:967885. doi: 10.3389/fmicb.2022.967885 (PMC9389584; doi:10.3389/fmicb.2022.967885)
Supplement: Supplementary file 3 [file Data_Sheet_3.zip › Figure S7.DOCX]

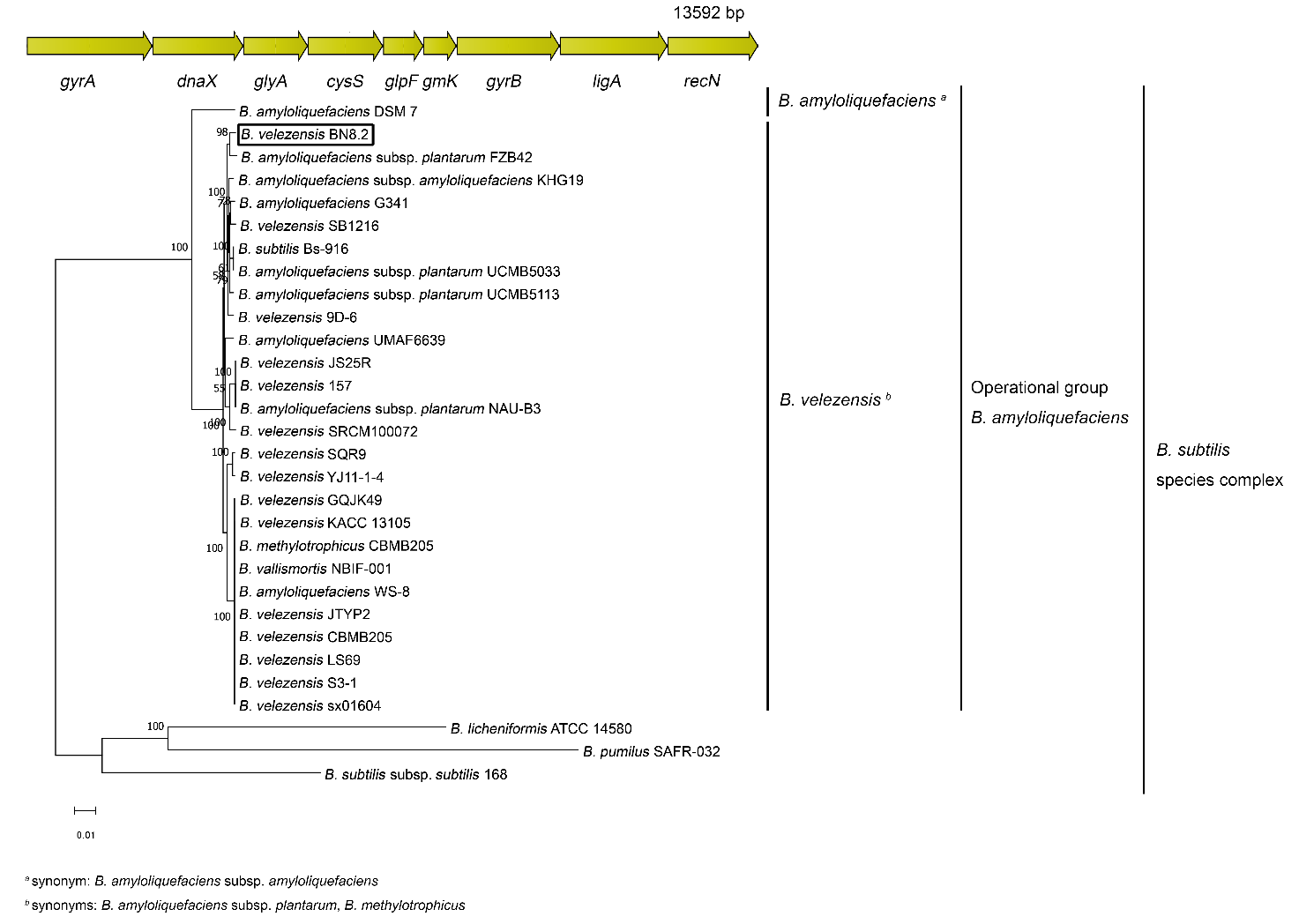


Figure S7. Neighbor-joining phylogenetic tree of 30 *Bacillus* spp. isolates constructed with the concatenated sequences of nine genes (DNA *gyrase subunit A* or *gyrA*, *DNA polymerase III* or dnaX, *serine hydroxymethyltransferase* or *glyA*, *cysteine-tRNA ligase* or *cysS*, *glycerol uptake facilitator protein* or *glpF*, *guanylate kinase* or *gmk*, *DNA gyrase subunit B* or *gyrB*, *DNA ligase* or *ligA*, and *DNA repair protein* or *recN*). Percentage of 1000 bootstraps are reported on the branch nodes. The bar below the tree represents the branch distance scale.
