# Supplementary material for: Designing a synthetic microbial community devoted to biological control: The case study of Fusarium wilt of banana
Source: Front Microbiol. 2022 Aug 5;13:967885. doi: 10.3389/fmicb.2022.967885 (PMC9389584; doi:10.3389/fmicb.2022.967885)
Supplement: Supplementary file 3 [file Data_Sheet_3.zip › Figure S8.DOCX]

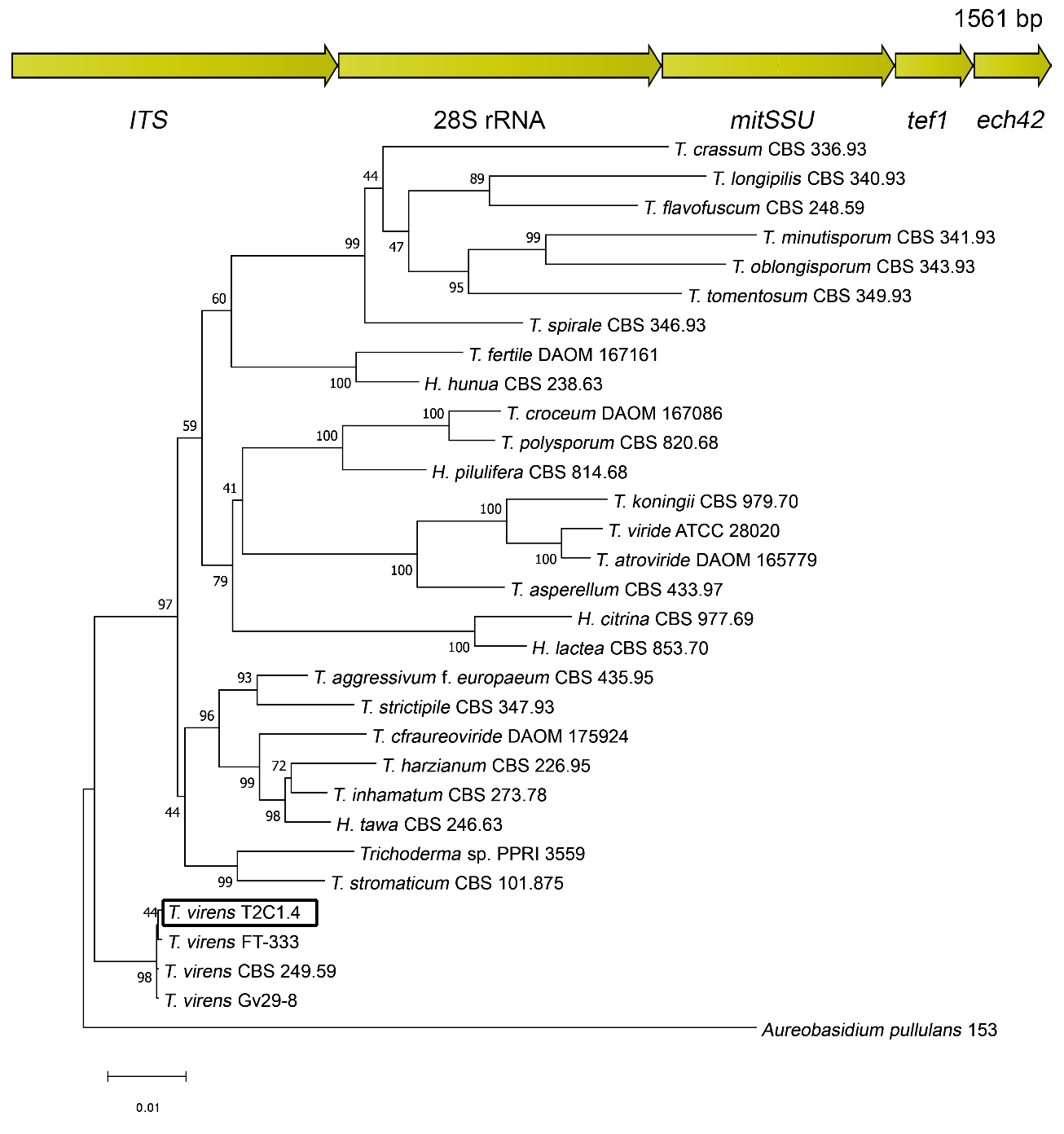


Figure S8. Neighbor-joining phylogenetic tree of 29 *Trichoderma* spp. isolates constructed with the concatenated sequences of five genes (*internal transcribed spacer* or *ITS*, 28S rRNA, *mitochondrial small subunit* or *mitSSU*, *translation elongation factor* 1α or *tef1*, and 42-kDa *endochitinase* or *ech42*). Percentage of 1000 bootstraps are reported on the branch nodes. The bar below the tree represents the branch distance scale.
